# Meta-Analysis of Smooth Muscle Lineage Transcriptomes in Atherosclerosis and Their Relationships to In Vitro Models

**DOI:** 10.20900/immunometab20210022

**Published:** 2021-05-21

**Authors:** Austin C. Conklin, Hitoo Nishi, Florencia Schlamp, Tiit Örd, Kadri Õunap, Minna U. Kaikkonen, Edward A. Fisher, Casey E. Romanoski

**Affiliations:** 1 The Department of Cellular and Molecular Medicine, The University of Arizona, Tucson, AZ 85721, USA; 2 The Cardiovascular Research Center, Division of Cardiology, Department of Medicine, NYU Grossman School of Medicine, New York, NY 10016, USA; 3 A. I. Virtanen Institute for Molecular Sciences, University of Eastern Finland, Kuopio 70211, Finland

**Keywords:** atherosclerosis, smooth muscle cells, macrophages, lineage tracing, single cell RNA-seq, cholesterol

## Abstract

**Background:**

Vascular smooth muscle cells (VSMC) exhibit phenotypic plasticity in atherosclerotic plaques, and among other approaches, has been modeled in vitro by cholesterol loading.

**Methods:**

Meta-analysis of scRNA-seq data from VSMC lineage traced cells across five experiments of murine atherosclerosis was performed. In vivo expression profiles were compared to three in vitro datasets of VSMCs loaded with cholesterol and three datasets of polarized macrophages.

**Results:**

We identified 24 cell clusters in the meta-analysis of single cells from mouse atherosclerotic lesions with notable heterogeneity across studies, especially for macrophage populations. Trajectory analysis of VSMC lineage positive cells revealed several possible paths of state transitions with one traversing from contractile VSMC to macrophages by way of a proliferative cell cluster. Transcriptome comparisons between in vivo and in vitro states underscored that data from three in vitro cholesterol-treated VSMC experiments did not mirror cell state transitions observed in vivo. However, all in vitro macrophage profiles analyzed (M1, M2, and oxLDL) were more similar to in vivo profiles of macrophages than in vitro VSMCs were to in vivo profiles of VSMCs. oxLDL loaded macrophages showed the most similarity to in vivo states. In contrast to the in vitro data, comparison between mouse and human in vivo data showed many similarities.

**Conclusions:**

Identification of the sources of variation across single cell datasets in atherosclerosis will be an important step towards understanding VSMC fate transitions in vivo. Also, we conclude that cholesterol-loading in vitro is insufficient to model the VSMC cell state transitions observed in vivo, which underscores the need to develop better cell models. Mouse models, however, appear to reproduce a number of the features of VSMCs in human plaques.

## INTRODUCTION

It has been long appreciated that vascular smooth muscle cells (VSMC) can exhibit phenotypic plasticity in vivo, especially in atherosclerotic plaques [1]. The establishment of model systems in vitro allowed mechanistically-oriented investigations of the phenotypic states observed in vivo [2]. Studies of arterial VSMC phenotypes tended to focus on two major states, named contractile and synthetic (e.g., ref. [3]). “Contractile” refers to the cells in the medial layer of a normal artery, which have the ability to adjust vessel tone in response to different stimuli. Under conditions of vascular pathology, such as atherosclerosis or injury (such as following an angioplasty), however, some cells in the media clonally proliferate and migrate to enter the intimal (subendothelial) space. These are the “synthetic” phenotype, so named because of their increased production, or synthesis, of extracellular matrix (ECM). Either in vitro or in vivo, the contractile machinery in synthetic cells is dampened, as indicated by the lower expression of multiple components, such as smooth muscle cell alpha-actin and myosin heavy chain. It has also been found in vitro and in vivo that when exposed to lipids or lipoproteins, VSMC could assume the appearance of a foam cell, typically identified by the accumulation of lipid droplets [4].

In 2003, it was reported that cholesterol-loaded VSMC foam cells not only had significant downregulation of contractile markers in vitro, but also had the induction of markers typically expressed by atherosclerotic plaque macrophages [5]. By using a combination of markers [6] or a proximity ligation assay [7] for human plaques, or lineage marking in mouse plaques [7,8], observations in vivo appeared similar to those in vitro, and suggested that a variant of the synthetic phenotype of arterial VSMC was a macrophage-like state. It became clear that the existence of such cells would have significant implications about the nature of the immune cell repertoire in atherosclerosis. Some estimates have reported that upwards of 30–40% [5–7] or even more [9] cells identified by conventional markers as macrophages in advanced mouse or human plaques were of VSMC origin. This has focused attention on the molecular features of these cells and their functional properties, especially in regard to plaque inflammation.

There has been an impressive growth in the number of papers exploring VSMC diversity in atherosclerosis, with a consequent acceleration of insights into what was known not only about the transition to a macrophage-appearing state, but also about the realization that VSMCs in plaques acquire features of cell types beyond what was previously described. While there is general agreement that VSMCs exhibit a higher degree of plasticity in atherosclerosis than previously appreciated, controversy lingers over the precise in vivo fate(s) of VSMC and their phenotypic states. Questions regarding specific cell states and fates of VSMCs remain despite the availability of VSMC lineage-traced mouse models and similar manipulations available to the field.

Thus, we felt it timely to assess the current state of the molecular understanding of VSMC phenotypic diversity by performing a meta-analysis of the available single cell transcriptomic data from VSMC lineage-traced cells from atherosclerotic mouse models. We were also particularly interested in the molecular features of VSMC-derived macrophage-like cells, not only to learn more about mechanisms for this transition, but also to infer their functional properties and contributions to plaque inflammation, given their aforementioned abundance in atherosclerotic arteries. Also, given the value of an in vitro system to perform intensive mechanistic investigations with potential in vivo relevance, we have also compared transcriptomic and epigenetic changes, which were induced in VSMCs by cholesterol-loading, to the emergent transcriptional profiles from our in vivo meta-analysis. Together with comparisons to single cell transcriptomics in human atherosclerotic lesions and in vitro macrophage signatures, we assess the fidelity of simple in vitro systems to model key aspects of more complex in vivo settings.

## MATERIALS and METHODS

### Murine Atherosclerosis Public Data Download and Integration

Count matrices from four different published single cell RNA sequencing (scRNA-seq) datasets were downloaded from the NCBI Gene Expression Omnibus (accessions listed in Supplementary Table S1) and then analyzed using Seurat version 4.0 [10]. Note that one study fit our other criteria for lineage mapped VSMCs in atherosclerotic lesions yet was not included based on data formatting [11]. Seurat objects were created from each dataset, and cells with <200 counts or >20,000 counts were removed. This is a quality control step, as it is thought that cells with high numbers of counts are more likely to be doublets (two cells caught in the same droplet), while cells with low numbers of counts are thought to be of poor data quality. Similarly, two additional quality control thresholds based on the methods of Alencar et al. [12] were implemented. For each cell, the percentage of counts that come from globin-encoding genes (including alpha, beta, and theta globin subunit genes) and mitochondrial genes was determined. Cells with >10% mitochondrial gene percent expression (which are thought to be of low quality, possibly due to membrane rupture) and cells with ≥5% percent globin gene expression (which are likely to be contaminating erythroblasts) were excluded. Data normalization, variable feature detection, feature scaling, and principal component analysis for 50 PCs were performed in Seurat using default parameters. The data were then normalized and integrated using Harmony [13] using the Seurat wrapper function RunHarmony; the group.by.vars parameter was set to each public dataset used in the analysis. Harmony embeddings were used in all relevant downstream analysis.

### Expression of ZsGreen Transcript in Pan et al.

Fastq files from Pan et al. [14] were downloaded from NCBI GEO using the SRA Toolkit and processed using cellranger count v6.0.0 and aligned to a custom reference mm10/ZsGreen-WPRE genome. The custom genome was constructed using cellranger mkref v6.0.0 on the mm10 reference genome v3.0.0 and the ZsGreen-WPRE coding sequence from an Ai6 construct. The sequence was taken from a plasmid sequence submitted by Hongkui Zeng (Addgene plasmid, Seattle, Washington, USA #22798) [15]. Following cellranger count, the filtered matrix data was analyzed in Seurat as described above.

### Single-Cell Clustering and Annotation

**Figure 1.**
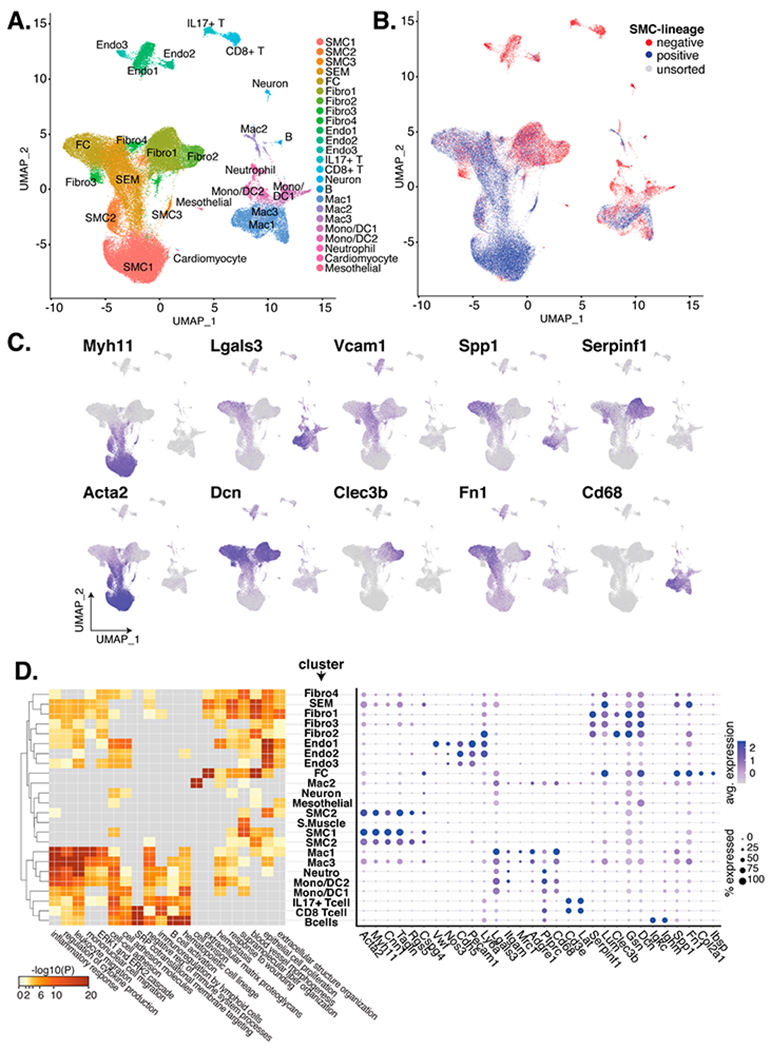
Meta-analysis of scRNA-seq on VSMC-lineage traced atherosclerotic lesions across 4 studies. (**A**) UMAP of single cells from 33 scRNA-seq samples across 4 studies are colored by cluster. (**B**) Cells are colored as VSMC-lineage positive (blue), as VSMC-lineage negative (red), or as unsorted cells (grey). (**C**) Marker expression for relevant cell types and UMAP locales. (**D**) On the left, clusters (y-axis) are paired to the negative log10 p-value from pathway enrichment analysis (x-axis). On the right, markers for VSMC (Myh11-Cspg4), endothelial cell (Vwf-Ly6a), myeloid cells/macrophages (Lgals3-Cd68), T-cells (Cd3e and Lat), fibroblasts (Serpinf1-Dcn1), B-cells (Igkc and Ighm), and fibrochondrocytes (Spp1-Ibsp) are shown by percentage of cluster cells (dot radius) and average expression (dot color).

Nearest neighbor detection and clustering were performed using the first 40 Harmony embeddings, followed by dimensional reduction to two dimensions using UMAP [16]. Canonical markers for endothelial cells, normal VSMCs, and fibroblasts were used to annotate relevant clusters (Figure 1). Annotation of fibrochondrocytes (FC) and SEM cells was done using previously reported markers [12,14,17] and evidence from relative UMAP positions. Annotation of leukocytes was done with published scRNA-seq markers [18]. Other immune cell types were annotated using Cluster Identity Predictor (CIPR) Shiny app web tool [19] with reference dataset ImmGen [20]. In order to achieve higher resolution annotation of immune cells, the Seurat object was subset to include only immune cell clusters; nearest neighbor detection, clustering, dimensional reduction, and cluster annotation were performed as described above.

### Marker Discovery and Differential Gene Expression

The Seurat functions FindMarkers and FindAllMarkers were used to detect cluster markers either between clusters or between each cluster and other cells, respectively. To remove potential false positive markers driven by different gene symbol annotations between datasets, we subset marker discovery to gene symbols with non-zero counts in all of the following publication’s data: Wirka et al., Pan et al., and Alencar et al. (Kim et al. was not included because only lineage positive cells were sequenced). This intersection of gene symbols was then passed to the FindMarkers and FindAllMarkers functions’ “features” parameter. To determine markers for lineage positive vs lineage negative cells within a cluster, we stratified cells using the “subset.ident” and “ident.1” parameters in FindMarkers.

### Single-Cell Data Trajectory Analysis

Single-cell trajectory analysis was performed using Monocle3 [21–23]. The Seurat object was subset to include VSMC-lineage positive cells. Following this, the VSMC-lineage positive data was converted into a Monocle3 object using the SeuratWrapper function as.cell_data_set. Following this, cluster_cells, learn_graph (use_partition = F), and order_cells were used to infer the trajectory between healthy VSMCs and VSMC-derived macrophages. Several root nodes were selected from within the normal SMC cluster and passed to order_cells.

### Single-Cell Clustering and Annotation of Human Carotid Artery Data

One scRNA-seq dataset (carotid 1) from Pan et al. [14] of human carotid atherosclerotic lesion cells from an endarterectomy sample was independently clustered and annotated as described above. Additional cluster annotation was performed using manual marker gene queries to the Human Protein Atlas [24].

### Data Visualization of scRNA-seq Data

Data visualizations of scRNA-seq data were performed using Seurat functions DimPlot, DotPlot, FeaturePlot, NNPlot, VlnPlot, VariableFeaturesPlot, and ElbowPlot. The Monocle3 trajectory was visualized using the Monocle3 function plot_cells (with trajectory graph shown and colored by pseudotime).

### Cell Culture and Cholesterol Loading In Vitro

Mouse VSMCs were isolated from thoracic aortas of 8–10 week-old C57BL/6 mice as described [5]. SMC lineage was confirmed by the presence of immunoreactivity for α-actin (Sigma, St. Louis, MO, USA) in >99% of the cells. Cells were grown in DMEM containing 10% FBS, 100 units/mL penicillin, and 100 ug/mL streptomycin. Cells with a passage number <5 were used in all described experiments. Cholesterol was delivered to VSMCs by using Chol:MβCD complex (Sigma, St. Louis, MO, USA, catalog #C4951). Sub-confluent VSMCs were incubated with Chol:MβCD (20 ug/mL) in 0.2% BSA for 24 or 48 h. Cells incubated with 0.2% BSA for 0, 24, or 48 h without Chol:MβCD treatment served as controls [5]. Chol:MβCD concentration was determined to be the maximum that would increase the cholesterol content of the cells (assessed by enzymatic kits as well as by oil red O staining) but would not cause toxicity for up to 72 h. These experiments were performed with approval from the Institutional Animal Care and Use Committee (IACUC) protocol IA16-00494 from NYU Langone Health (New York) approved on 07/31/2017.

Mouse immortalized aortic VSMC cell line (MOVAS, purchased from ATCC, Manassas, VA, USA) was cultured in DMEM supplemented 10% FBS, 100 U/mL penicillin, 100 μg/mL streptomycin and 200 μg/mL geneticin, and incubated at 37 °C in 5% CO_2_ environment. Twenty-four hours before cholesterol loading, the culture medium was replaced with DMEM supplemented with 0.02% BSA. Subsequently, the medium was replaced with DMEM supplemented with 0.02% BSA and Chol:MβCD (Sigma, St. Louis, MO, USA, catalog #C4951) for 72 h, after which the cells were collected for scRNA-seq. Approximately 2000 cells treated with 0, 25, 50 and 100 μg/mL cholesterol were pooled, yielding a total of approximately 8000 cells for 10× Genomics Chromium instrument loading. Cell viability prior to loading was >85% for all treatments, as measured by hemocytometry with Trypan blue staining.

### Transcriptional and Epigenetic Profiling

RNA was extracted from cultured VSMCs using the Quick-RNA Micro Prep kit from ZymoResearch (Irvine, CA, USA, #R1051), including optional DNase I treatment. mRNA was selected through poly-A isolation using Oligo d(T)25 beads (New England BioLabs, Ipswich, MA, USA, #S1419S). Selected RNA was fragmented, followed by single strand cDNA synthesis using a Superscript III First-Strand Synthesis System (ThermoFisher Scientific, Waltham, MA, USA, #18080051), followed by second strand synthesis using DNA Polymerase I (Qiagen/Enzymatics, Beverly, MA, USA, #P7050L). dsDNA ends were repaired with T4 DNA Polymerase (Enzymatics, Beverly, MA, USA, #P7080L). Barcode adapters (BIOO Scientific NEXTflex, Austin, TX, USA, #514104) were ligated onto the ends of sequences using T4 DNA Ligase (Enzymatics, Beverly, MA, USA, #L-6030-HC-L) and samples were treated with Uracil DNA Glycosylase (UDG) (Enzymatics, Beverly, MA, USA, #G5010L). Libraries were then amplified by PCR (Phusion Hot Start II, ThermoFisher Scientific, Waltham, MA, USA, #F549L) and purified (ZymoResearch, Irvine, CA, USA, #D5205) for high-throughput sequencing.

ChIP-seq was performed using an H3K27ac antibody (abcam, Cambridge, MA, USA, #ab4729 lot GR45787-1) as previously described [25]. Briefly, cells were fixed at room temperature with 1% paraformaldehyde in PBS for 10 min, and then quenched with glycine. Between 3 and 5 million cells were used for each ChIP. Fixed lysates were sonicated using a BioRuptor Standard (Diagenode, Denville, NJ, USA), and then immunoprecipitated using antibodies bound to a 2:1 mixture of Protein A Dynabeads (Invitrogen, Waltham, MA, USA, #10002D) and Protein G Dynabeads (Invitrogen, Waltham, MA, USA, #10004D). Following immunoprecipitation, crosslinking was reversed, and libraries were prepared using the same method described for RNA-seq beginning with dsDNA end repair and excluding UDG. For each sample condition, an input library was also created using an aliquot of sonicated cell lysate that had not undergone immunoprecipitation. These samples were sequenced as below and used as background during peak calling.

### scRNA-seq Library Preparation, Sequencing and Data Processing for MOVAS Cells

scRNA-Seq was carried out using the Single Cell 3’ Reagent Kit (v3 Chemistry; 10× Genomics) following the manufacturer’s protocol. The library was sequenced in an Illumina NextSeq instrument using the cycling program Read1: 28 bp, Index1: 8 bp, Read2: 91 bp. The cellranger count pipeline (version 3.0.2; 10× Genomics) and the mm10 reference package (version 3.0.0; 10× Genomics) were used to process the sequence read files, including genome alignment, UMI deduplication, transcript counting, cell barcode attachment and cell calling steps. Downstream processing was performed using Seurat (version 3.1.0 [26]) in R (version 3.5.3). For cell quality filtering, cells with 2500–7500 genes detected, 5000–25,000 total UMI-s and <7.5% mitochondrial reads were retained. This resulted in approximately 4300 cells, with a median of 4600 genes and 15000 UMI-s per cell. The RNA counts were processed using the standard scRNA-Seq workflow recommended by the authors of Seurat v3 with a clustering resolution parameter of 1.0. Cluster markers were calculated using the Wilcox test, requiring expression in at least 10% of cells in the cluster and a fold change of at least 0.25. Cluster markers with a positive fold change were used for gene ontology enrichment analysis with the gProfiler web tool (database release 2020-07-22 [27]) using all GO Biological Process gene sets. Gene categories were filtered to exclude unenriched (FDR>5%) and very large (>1000 genes) categories, and GO semantic similarity filtering (Schlicker’s relevance >0.5 [28]) was used to reduce the lists (GOSemSim R package version 2.8.0 [29]). For each cluster, up to 7 enriched categories were selected by *p* value, and the resulting categories were plotted for all clusters.

### Sequencing Data Samples, Mapping, and Normalization

Libraries were sequenced on an Illumina HiSeq 4000 according to manufacturer’s specifications at the University California San Diego and at the University of Chicago. Reads from ChIP-seq experiments were mapped to the mm10 build of the mouse genome with Bowtie2 [30] and RNA-seq reads were mapped to mm10 with STAR [31]. Mapped reads were organized in HOMER [32] using its preferred data structure using the makeTagDirectory function. Expression matrices were calculated in HOMER using the analyzeRepeats function counting tags in the mm10 RefSeq gene body annotations with either no normalization, for input into DESeq2, or with-rpkm, for visualization in heatmaps. Because of low read counts (<1 million) and failure to cluster with replicates, control 48hr replicate 1 was discarded from all downstream analysis.

### RNA-seq Analysis

RNA-seq analysis was performed in R (V4.0.2) using DESeq2 package v1.28.1 [33]. Only genes with more than 70 raw counts in at least one sample were kept. Principal Coordinates Analysis (classical multidimensional scaling) was performed using cmdscale() function on log2 transformed normalized counts obtained with the DESeq2::estimateSizeFactors() function. Differential Expression analysis was done by comparing the full model “~time + treatment:time” against the reduced model “~time” using a likelihood ratio test (LRT), giving 4143 genes with an adjusted *p*-value <0.05. These 4143 DE genes were clustered with the pheatmap package (v1.0.12) using “correlation” clustering distance after row *Z*-scaling of normalized counts, and genes were split into their main four clusters by cutting the dendrogram tree row with cutree() at *k* = 4. We then further filtered each cluster by only keeping genes with an absolute log fold change of 0.6 or larger in either the 24 or 48 h pairwise contrast between cholesterol and control samples. This resulted in a total of 1702 genes with both *p*_adj_ < 0.05 and |logFC| ≥ 0.6 across all 4 clusters (C1 = 684, C2 = 264, C3 = 225, C4 = 529), also visualized as volcano plots generated with ggplot package (v3.3.2). Next, we calculated the mean of mean-centered and variance-scaled normalized counts for the genes in each of the clusters at every control and treatment time point, as performed previously [34]. Heatmaps of Cholesterol Biosynthetic and Unfolded Protein Response genes were made using the R package “gplots” heatmap.2 function. Genes sets for all 4 clusters were input in separate analyses using Ingenuity Pathway Analysis (Qiagen) “Core Analysis” option.

### ChIP-seq Analysis

ChIP-seq H3K27ac-defined genomic regions were identified relative to un-immunoprecipitated, fixed chromatin, or “input”, as a negative control. Peaks were called using HOMER with the findPeaks program using the - histone option. Differential peaks between experiments were determined using the getDifferentialPeaks program with default parameters (normalized tag count difference >4 fold and poisson enrichment *p*-value < 0.0001). Peak merging was performed in HOMER using the “mergePeaks” program to define the union of H3K27ac regions. Each region was centered on the calculated greatest Nucleosome Free Region (NFR) using the -nfr option. The center of the NFR generally has very few H3K27ac tags because this corresponds to the location of TF binding and histone/nucleosome exclusion. In Figure 5B, the central 200 bp sequences of all promoter distal H3K27ac regions (defined as > 3kb from promoter start sites using RefSeq annotations in getDistalPeaks.pl in HOMER) were input for de novo motif analysis using HOMER’s “findMotifsGenome.pl” program. GC-matched 200 bp background sequences are sampled from the genome and used as background for enrichment analysis. Differential H3K27ac regions in cholesterol loading relative to control were defined using “getDifferentialPeaks.pl” with the 24 h cholesterol loaded sample as the foreground and the 24 h control as the background. Motif finding was calculated using the central 100 base pair sequences for cholesterol-up-regulated regions as the foreground and control-up-regulated sequences as the background. Histograms of motif frequency were calculated in annotatePeaks.pl using the -m and -hist options, which output the frequency (y-axes) relative to the center of the NFR (0 bp on x-axes).

### Comparison of in vivo scRNA-seq Data to In Vitro Bulk RNA-seq and In Vitro Microarray Data

Meta-analyzed murine scRNA-seq data and in vitro cholesterol treated murine VSMC bulk RNA-seq data were compared along with five other publicly available datasets (described in Supplementary Table S1). Gene expression values for each cell cluster were produced using the AverageExpression function in Seurat (which exponentiates log data, therefore output is depth normalized in non-log space); gene symbols were again filtered to included symbols at the intersection of three publications’ data (see explanation above in “Marker Discovery/Differential Gene Expression”). Following this, mean expression values for replicate experiments were calculated; two replicates from the in vitro RNA-seq data were not included (control 48 h replicate 1, as noted above, and control 0 h replicate 1) because of their failure to cluster with other replicates of the same condition. Hierarchical clustering of each experimental observation or cell cluster was then performed using the scipy.clustering.hierarchy function linkage (with method = “complete” and a user defined distance metric) in python(v3.8.5). Spearman correlation was used as the distance metric (1 minus Spearman correlation co-efficient); we reasoned that a non-parametric correlation metric should be robust to differences in distributions between the datasets. Sample clustering was performed using the top 2000 most variable genes in the meta-analyzed mouse in vivo data (this number was pre-specified before our analysis based on the default argument of Seurat’s FindVariableFeatures). Only genes/orthologs measured across all platforms were used for pairwise distance calculations, which involved excluding some of the 2000 most variable genes. For bulk RNA-seq VSMC samples, expression values were normalized using RPKM. oxLDL and M1/M2 macrophage bulk RNA-seq data were analyzed using reported expression values (TPM and featureCounts, respectively). Human/mouse orthologs were determined using gene symbol sharing. Dendrogram figures were generated using the scipy.clustering.hierarchy dendrogram function, and heatmaps plots were generated using pandas.DataFrame.corr (method = “spearman”) and the seaborn (version 0.11.1) clustermap function.

## RESULTS

### Meta-Analysis of VSMC Lineage Traced scRNA-seq Datasets

To gain insight into the diversity of VSMC-derived cell types in murine arterial plaques, and to evaluate their relationships to an in vitro system, we analyzed public data from four recent publications that utilized VSMC lineage tracing in mouse models of atherosclerosis [12,14,17,35] (Table 1). Criteria for inclusion included the availability of scRNA-seq (scRNA-seq) data that were generated from arteries over a time course of high fat feeding in *Apoe^−/−^* or *Ldlr^−/−^* (also called *Apoe* and *Ldlr* knock out (KO)) mice whose VSMCs were lineage traced through tamoxifen induction of Cre-ERt downstream of the Myh11 promoter that ultimately causes a fluorescent protein to be permanently expressed in lineage positive cells [36]. To generate a normalized and integrated dataset for meta-analysis, we employed Harmony [13] on over two dozen different experimental conditions including FACS-sorted VSMC lineage traced cells, lineage negative cells, and unsorted cells (Supplementary Table S1). This resulted in a dataset of >70,000 cells after quality control filtering (METHODS). Clustering of cells in Seurat [10] resulted in 24 different clusters. We visualized the integrated data using uniform manifold approximation and projection [16] (UMAP) for dimensionality reduction (Figure 1A). Cells in the UMAP were colored as VSMC lineage positive, negative, or unsorted based on the relevant experiments from the included datasets (Figure 1B). We annotated the clusters using cell type markers available defined in previous reports, using CIPR [19], and using a recent meta-analysis of leukocytes in atherosclerosis [14,18]. This led to the following cluster designations: 3 SMC clusters, 1 SEM cluster (stem-cell, endothelial cell, monocyte) that has been proposed to represent an intermediate VSMC phenotypic switching state [14], 1 fibro-chondrocyte (FC) cluster, 4 fibroblast (fibro.) clusters, 3 endothelial (endo.) cell clusters, 2 T-cell clusters (one IL17^+^ and one CD8^+^), 1 B-cell cluster, 3 macrophage clusters, 2 monocyte/dendritic (mono/DC) cell clusters, 1 neutrophil (neutro.) cluster, and 3 other clusters of various cells (neurons, striated muscle, and mesothelial, which was annotated based on its expression of Msln and Upk3b [37]) (Figure 1A). We find that the greatest proportion of VSMC lineage-positive cells reside in the SMC, SEM, and FC clusters with notable representation in the macrophage clusters as well.

We plotted the expression of several marker genes in Figure 1C, including myosin heavy chain (Myh11), whose promoter sequence is used to drive the VSMC lineage tracing. As previously shown, Myh11 expression is most prevalent in the VSMC clusters with decreasing expression into the SEM locale where Vcam1 is characteristically expressed. Expression of Acta2 (smooth muscle cell alpha-actin) shows a similar pattern as Myh11. Macrophage-related markers Lgals3 and Cd68 are most highly expressed in the macrophages, though Lgals3 is also expressed in the FCs and SEMs. Additional markers are expressed in various combinations of cell types with FCs and Fibros, including Spp1 (FC and Macs), Serpinf1 (Fibros), Dcn (Fibros and FCs), Clec3b (Fibros) and Fn1 (SEMs, FCs, Fibros, and Macs). While few of these transcripts define absolute boundaries between clusters, their distributions remain useful for interpreting cell state and fate. More quantitative representations are shown in Figure 1D for these and additional transcripts. Marker genes for cell clusters are recorded in Supplementary Table S2.

Next, we submitted the top 100 differentiating transcripts per cluster (versus all other cells, sorted by ascending *P*-value) to the pathway analysis enrichment program Metascape [38]. The 20 enriched terms are shown in Figure 1D. As expected, immune cell clusters were highly enriched in immune-related ontologies such as ‘inflammatory response’ and ‘regulation of cytokine production’. Non-immune cell cluster enrichments included ‘blood vessel morphogenesis’ (SMCs/FCs/Endos/Fibros), and ‘extracellular matrix proteoglycans’ (FCs). Interestingly, the macrophage cluster Mac2 was highly enriched for the ‘cell division’ ontology, indicating this population to be highly proliferative.

### Transcriptome-Based Cell Type Characterization Is Variable across Five Studies

**Figure 2.**
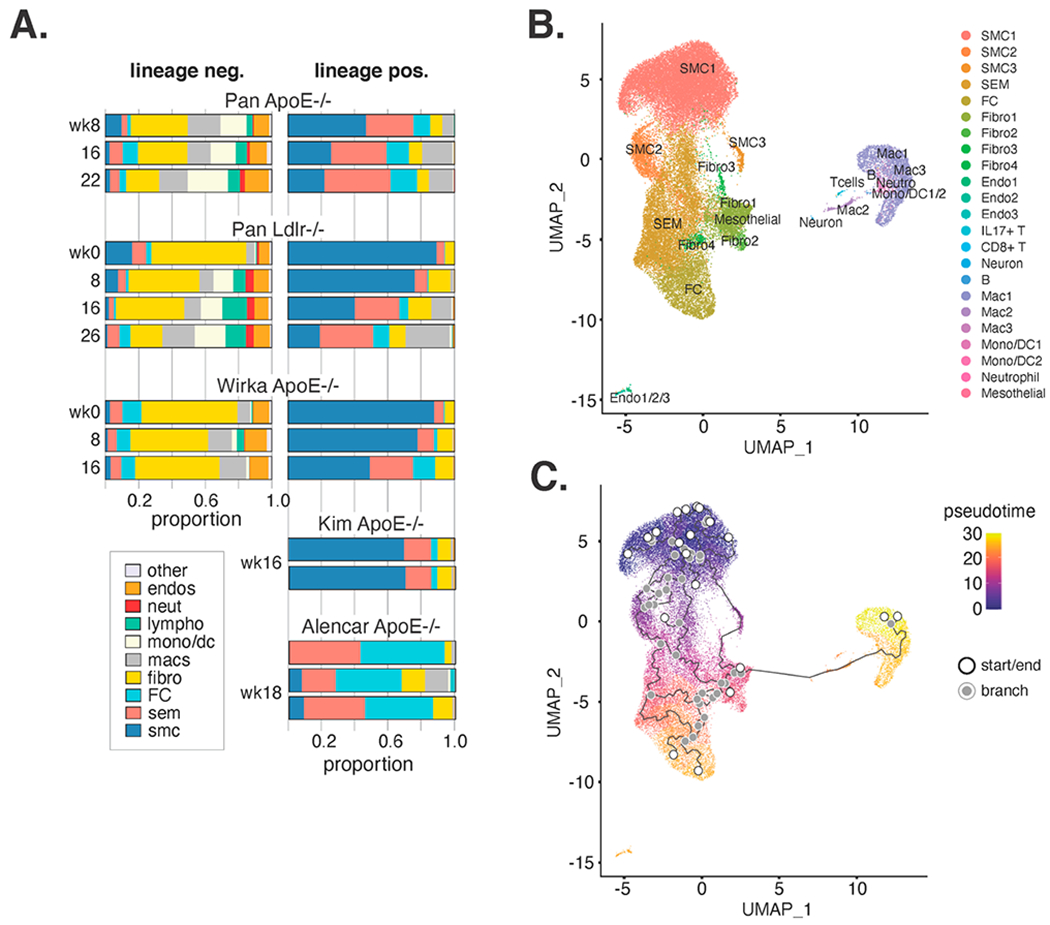
Proportions of VSMC-lineage traced and untraced cell populations vary by study and time on pro-atherogenic diet. (**A**) Relative proportions of VSMC-lineage sorted negative (**left**) and positive (**right**) cells are shown by publication and time on pro-atherogenic diet. Note that Alencar et al. microdissected BCA lesions whereas others dissected lesioned portions of arteries. (**B**) SMC-lineage positive cells only were re-visualized by UMAP and colored according to cluster annotations in Figure 1. (**C**) Pseudotime analysis in Monocle3 results are shown for lineage positive cells according to UMAP in B.

Given the presence of VSMC lineage positive cells across multiple clusters in our meta-analysis, we next sought to identify patterns of transcriptionally defined populations of VSMC positive and VSMC negative cells in atherosclerotic lesions over time and across studies. Five datasets (Table 1) in our meta-analysis were fit for this analysis, including two from Pan. et al. [14]: one with *Apoe^−/−^* mice fed a HFD (high fat diet; synonymous WD, or western diet herein) for 8, 16, and 22 weeks, and another with *Ldlr^−/−^* mice for 0, 8, 16, and 26 weeks on an HFD. The third was reported by Wirka et al. [17] using *Apoe^−/−^* mice with HFD for 0, 8, and 16 weeks. The fourth and fifth datasets did not evaluate VSMC lineage negative cells, and were excluded from this analysis. Clusters were combined for each cell type (i.e., SMC1, SMC2, and SMC3 became SMC) and proportions were computed for both VSMC lineage positive and lineage negative cells (Figure 2A).

The primary observation from these data was the considerable variability across studies, which was further confirmed when looking at each sample individually (Supplementary Figure S1; origin of samples in Supplementary Table S1). In particular, proportions of VSMCs and macrophages were most variable with relatively more VSMCs, and fewer macrophages, observed in Wirka and Kim datasets relative to Pan and Alencar.

We sought to determine whether auto-fluorescence could explain the high prevalence of lineage positive macrophages in the data from Pan et al., so we downloaded their raw sequencing data and mapped it to a custom mm10 genome with the ZsGreen1-WPRE sequence from the Cre-inducible Ai6 reporter [15]. We find that expression of ZsGreen transcripts are highly variable between cell type clusters; we also find that ZsGreen transcript is consistently higher in lineage-positive sorted cells compared to lineage-negative cells of the same cluster (Supplementary Figure S2). This result suggests that lineage positivity of sorted macrophages is unlikely to be the result of auto-fluorescence.

Despite these differences, we found that the proportion of ‘contractile’-like VSMCs consistently decreases with weeks on HFD, whereas proportions of SEMs and macrophages increase. Together, the high degree of variability in cell proportions for atherosclerotic scRNA-seq data raises questions as to the sources of this variation.

### Analysis of only VSMC Lineage Positive Cells Discloses a Trajectory Linking VSMCs to Macrophages

Over 40 thousand VSMC lineage positive cells were present in this meta-analysis, which we reasoned would allow us to make inferences about the re-differentiation trajectories of VSMCs in atherosclerosis. Therefore, we submitted only VSMC lineage positive cells to Seurat’s dimensionality reduction and visualization (Figure 2B). We found that the SMC/SEM/FC/Fibro clusters remained adjacent to one another, whereas cells in macrophage clusters Mac1 and Mac3 were now adjacent to cells in Mac2 that stretched toward the SMC/SEM/FC/Fibro clusters along UMAP axis 1 (Figure 2B). We applied pseudotime analysis in Monocle3 [21–23], which resulted in many trajectories originating in the SMC1 cluster. Interestingly, one trajectory included a link connecting the Fibro1 cluster to Mac2 and concluding in Mac1 (Figure 2C). Notably, several trajectories were drawn among non-macrophage clusters with many traversing the SEM population. This result is consistent with the proposed model that SEMs are a de-differentiated VSMC state from which other VSMC-derived cell types arise [14]. Interestingly, we find that the Mac2 cells are enriched in pro-proliferative transcripts, including Plk1, Birc5, and Ccna2 (Figure 1D and Supplementary Figure S3). This may represent a transitory proliferative VSMC phenotype that occurs upon trans-differentiation to a macrophage-like state. We sought to further investigate the hypothesis that VSMCs transdifferentiate into macrophages first through an SEM phenotype and then through a fibroblast-like phenotype followed by a proliferative macrophage-like state. For each cell in the Mac2 cluster, we visualized the twenty nearest neighbors to that cell in high dimensional space (the Harmony embedding). We find that many nearest neighbors of Mac2 fall in the other macrophage clusters (consistent with our hypothesis), and many nearest neighbors of Mac2 are scattered across the SMC, SEM, FC, and Fibro clusters, which is inconsistent with our hypothesis; specifically, it demonstrates that the trajectory through a fibroblast-like state is likely an oversimplification resulting from the dimensionality reduction. Thus, we conclude that while Mac2 may represent a phenotypic intermediate between VSMCs and VSMC-derived macrophages, it is unclear what other intermediate states a VSMC must progress through to re-differentiate into a macrophage.

### Re-Clustering of Immune Cells Shows Cellular Subtypes and Differential Expression between Lineage Positive and Lineage Negative Macrophages

**Figure 3.**
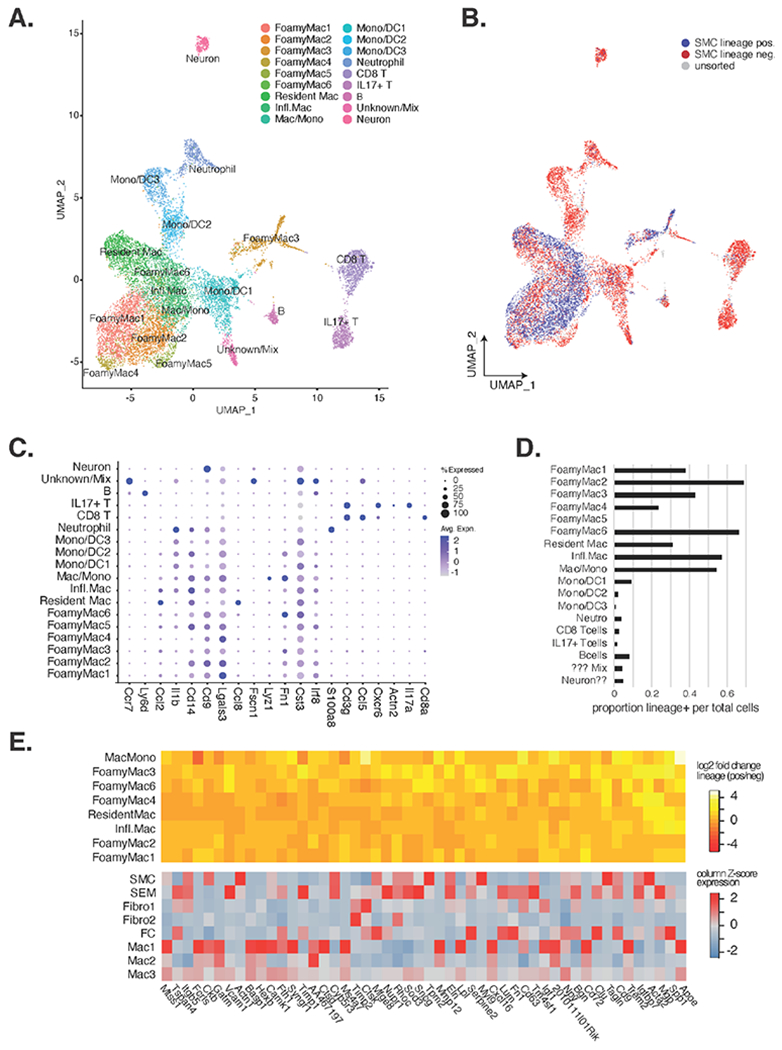
Further analysis of immune cells reveals 18 clusters with VSMC lineage positive cells most closely resembling macrophage subsets. (**A**) UMAP reduction and clustering of only immune clusters from Figure 1 found 18 clusters that were annotated by leukocyte markers in Zernecke et al. (**B**) SMC lineage positive cells are shown in blue, lineage negative in red, and unsorted cells in grey. (**C**) Dotplot depiction of key immune cell marker genes. (**D**) Proportion of lineage positive cells (x-axis) by immune cell cluster (y-axis) are shown. (**E**) The top heatmap shows the log2 fold change of VSMC lineage positive over lineage negative expression of transcripts (x-axis) in the different immune cluster (y-axis) for the 50 most up-regulated genes in lineage positive cells. The bottom heatmap shows the relative expression of these same genes (x-axis) for mouse atherosclerotic lesions (unit = *Z*-scores for each transcript across clusters).

We next reasoned that re-clustering and re-visualizing non-SMC/SEM/FC/Fibro/EC cells could provide better resolution of immune cell states. The UMAP from this analysis, from 13.4 thousand cells in Figure 3A consisted of 18 cell clusters with the following annotations derived from marker genes reported by the recent meta-analysis of leukocytes in murine atherosclerosis [18]: 6 Trem^2+^ Foamy Macrophage clusters (FoamyMac1-6), 1 resident macrophage cluster, 1 inflammatory macrophage cluster, 1 macrophage/monocyte mixed cluster, 3 monocyte and dendritic cell mixed clusters (mono/DC1-3), 1 neutrophil cluster, 1 CD8^+^ T cell cluster, 1 IL17^+^ T cell cluster, 1 B cell cluster, and 2 other stromal clusters (Figure 3A). Marker genes for the re-clustered immune cells are displayed in Supplementary Table S3. The comparison of VSMC lineage positive to negative cells in this UMAP supported that VSMC positive immune-like cells are most similar transcriptionally to macrophages because they align with those clusters (Figure 3B). Using reported markers from a recent meta-analysis of leukocytes in atherosclerosis, we annotated each immune population [18]. Discriminating markers from this analysis are shown in Figure 3C. We found VSMC lineage positive cells were most prevalent in foamy macrophage clusters (between 23–68% of cells in 5 of 6 foamy macrophage clusters; Figure 3C,D). We also found VSMC lineage positive cells composed more than 50% of inflammatory macrophages, cells in the mono/CD cluster, and greater than 30% of resident macrophages. Next, to ascertain which transcripts were most specific to VSMC lineage positive versus negative cells in these clusters, we identified the top 50 most up-regulated genes (Figure 3E; top heatmap colors are log2 fold changes of lineage pos versus lineage neg). We next queried which in vivo clusters these genes are abundantly expressed in and found that they are generally highly expressed in one of a variety of cell types within the atherosclerotic plaque, including SMCs, SEMS, FC and macrophages (Figure 3E, bottom). Notably, Acta2 is more highly expressed among lineage positive macrophages than among lineage negative macrophages. Considerable variability in lineage positive cells per cluster is observed across individual samples and studies (Supplementary Figure S4), making absolute quantification of lineage positive cells challenging.

### Widespread Transcriptional Changes Are Induced by VSMCs Cholesterol Loading In Vitro

**Figure 4.**
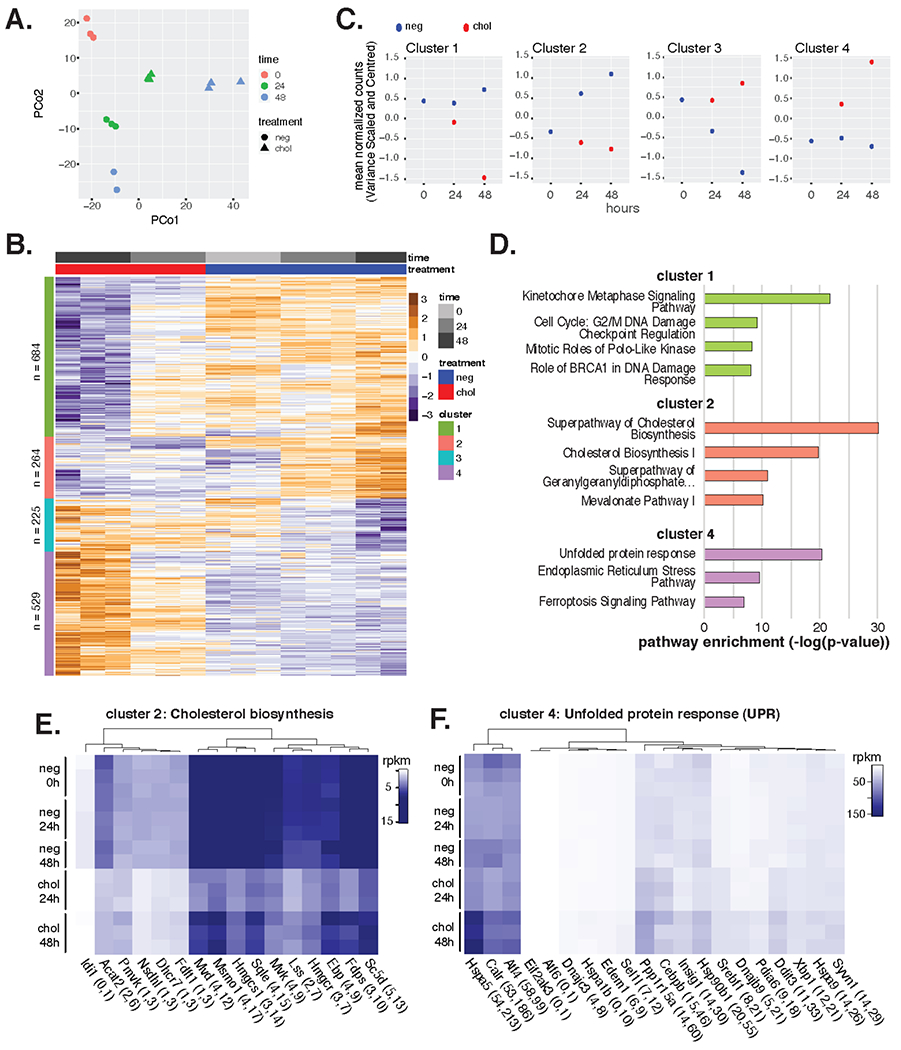
Primary cultured mVSMCs exhibit widespread transcriptional changes upon cholesterol loading. (**A**) Principal Coordinates (PCo) Analysis of mVSMC RNA-seq samples ± cholesterol at 0 h, 24 h, and 48 h timepoints. (**B**) Transcript expression values per condition are shown by heatmap as normalized per row with cluster designation along left side bar. (**C**) Mean expression of transcripts per gene set over time. (**D**) Most enriched pathways are shown from Ingenuity Pathway Analysis. None were significant for cluster 3. (**E**) Cluster 2 Cholesterol Biosynthetic Pathway transcript expression across conditions. (**F**) Cluster 4 Unfolded Protein Response transcript expression across conditions.

To model gene expression changes consequent to high cholesterol exposure as occurs in atherosclerotic lesions, we exposed murine VSMCs isolated from aortic segments of C57BL6/J mice to 20 ug/mL cyclodextrin-cholesterol complexes or control (0.2% BSA) for 0, 24, and 48 h in vitro (METHODS, “Cell culture and cholesterol loading in vitro”) RNA sequencing (RNA-seq) was performed on biological triplicates yielding an average of 21.46 million mapped reads per sample following the removal of one sample for low read count (<1 million, control 48 rep1) (Supplementary Table S4). To explore the unbiased global expression changes, we performed Principal Coordinates (PC) Analysis and observed tight concordance between replicates of the same condition (Figure 4A). The 1st PC corresponded with duration of cholesterol loading and the 2nd PC diverged with time. Differential expression analysis identified 4143 differentially expressed genes across the dataset at a False Discovery Rate (FDR) [39] of 5%. Together, these findings demonstrate that exposure of VSMCs to a cholesterol-rich environment reorganizes their transcriptional program.

To better understand the biological pathways regulated by cholesterol in VSMCs, we utilized the hierarchical relationship among differential gene profiles to identify distinct patterns of gene expression. This resulted in 4 regulatory profiles, or clusters (Figure 4B,C; Supplementary Figure 5SA). Cluster 1 transcripts (*n* = 684) summarizes a profile of stepwise down-regulation by cholesterol loading compared to control (Figure 4C). Pathway analysis found this gene set to be significantly enriched for kinetochore metaphase signaling pathway genes, as well as genes involved in the G2/M DNA damage cell cycle checkpoint (Figure 4D; green bars). Cluster 2 transcripts (*n* = 264) fit a pattern whereby time in control media up-regulates expression and exposure time to cholesterol down-regulates expression (Figure 4C). This gene set is highly enriched in members of the super-pathway of cholesterol biosynthesis that is known to be inhibited by high extracellular cholesterol levels [40,41]. Expression for genes in this pathway is visualized by heatmap in Figure 4E, and with respect to their location in the metabolic pathway in Supplementary Figure 5SB. Notably, cholesterol loading down regulated nearly every enzyme in the cholesterol biosynthetic pathway in vitro. The behavior of transcripts in cluster 3 (*n* = 225) is described by progressive down-regulation in control media; however, this set is not enriched in any pathways tested. Lastly, transcripts in cluster 4 (*n* = 529) exhibit progressive up-regulation by cholesterol over time relative to controls (Figure 4C). This gene set is enriched in the Unfolded Protein Response (UPR) pathway. Expression profiles across conditions of UPR genes are shown by heatmap in Figure 4F and in network format in Supplementary Figure 5SC. Notably, several Transcription Factors (TFs) in the UPR are up-regulated by cholesterol including members of all three branches of the UPR: Atf4, Atf6, and Xbp1 (Figure 4F).

### Cholesterol Loading of VSMCs In Vitro Modestly Reorganizes the Active Epigenetic Landscape

**Figure 5.**
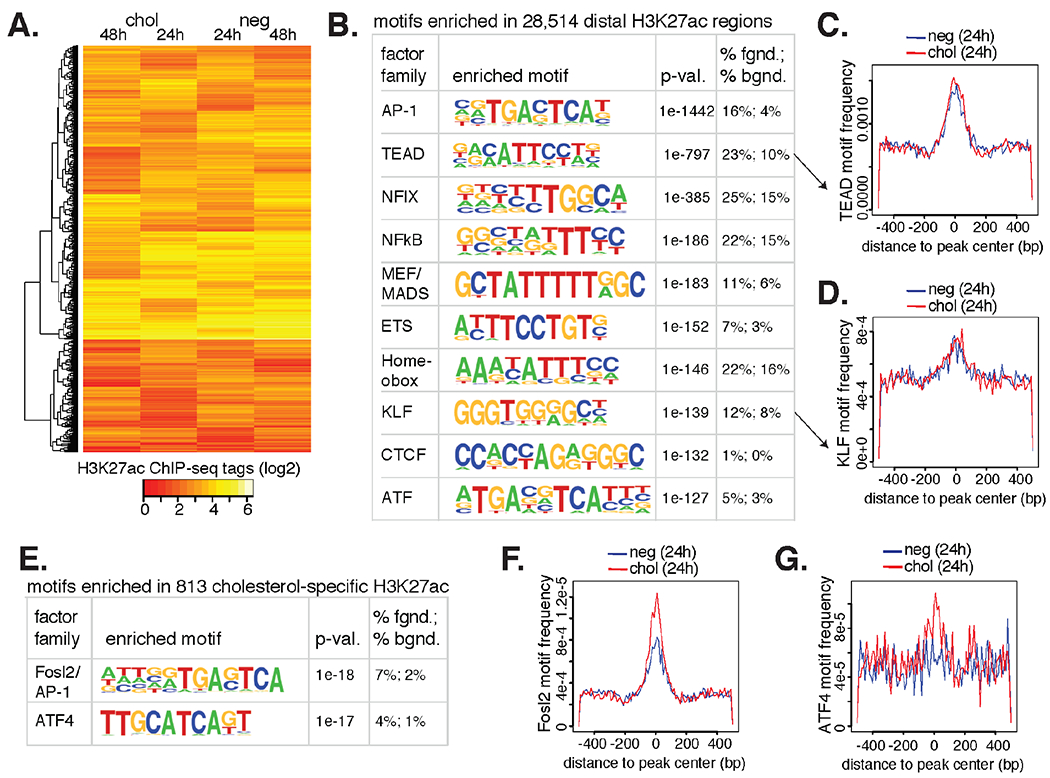
48 h of mVSMCs cholesterol loading in vitro has a subtle effect on histone acetylation. (**A**) The top 5000 most variable H3K27ac gene-distal regions (>3 kb from TSS) across experimental conditions. (**B**) De novo motif analysis of combined H3K27ac+ regions are shown with predicted factor family TFs, enrichment *p*-value, and percent occurrence in the data (fgnd) compared to random GC-match genome (bgnd). (**C**) Frequency of the TEAD motif (y-axis) is plotted relative to the center of H3K27ac+ nucleosome free peak centers (x-axis). (**D**) The enriched KLF motif is shown as in C. (**E**) Fosl2/AP-1 and ATF4 motifs are more enriched at H3K27ac+ regions in cholesterol loaded VSMCs relative to the negative (neg) control. (**F**) The Fosl2 motif is more frequent in cholesterol H3K27ac+ peak centers relative to neg. (**G**) The ATF4 motif is more frequent in cholesterol H3K27ac+ peak centers relative to neg.

To gain insight into how cholesterol reorganizes the epigenetic landscape in mVSMCs we performed Chromatin Immuno-Precipitation with high-throughput sequencing (ChIP-seq) for histone H3 acetylation on lysine 27 (H3K27ac), which is a post-translational modification present on nucleosomes surrounding active regulatory elements, including promoters and enhancers [42]. An average of 19.9 million mapped sequence tags were analyzed for the 24 h and 48 h control and cholesterol conditions in the same experimental in vitro model as for transcriptomics (Supplementary Table 4S). Analysis of the top 5000 most variable H3K27ac-marked loci are shown in Figure 5A, where we generally did not observe large changes in the amount of this epigenetic mark between control and cholesterol conditions. We therefore submitted the union of these datasets (*n* = 28,514; 200 bp sequences) to de novo motif finding analysis to return the TF motifs that are utilized in this cultured VSMC model. The top ten most enriched motifs are shown in Figure 5B, topped by the AP-1 and TEAD motifs. Notably, the frequency of these motifs in their genomic context were greatest near the center of the nucleosome-free regions as defined by H3K27ac ChIP-seq, consistent with their cognate TFs binding to these peak centers (Figure 5C,D). Notably, TEAD and KLF motif frequencies were similar in control and cholesterol-treated conditions, suggesting proteins of these TF families bind DNA in both conditions.

Although we observed little re-distribution of H3K27ac signal upon cholesterol exposure, 813 regions, representing 1.6% of all H3K27ac regions identified after cholesterol exposure, were differentially enriched for two motifs when compared to control (Figure 5E). These motifs were for Fosl2 (a member of the AP-1 family), and ATF4 (a member of the ATF/CREB family). Albeit modest, frequencies for each of these motifs were greater in H3K27ac regions defined with cholesterol-loading compared to control (Figure 5F–G). Extracellular matrix and actin-based processes were enriched pathways for the genes nearest to H3K27ac regions containing ATF4 and Fosl2 motifs (Supplementary Table 5S). These data are consistent with induction of the UPR by cholesterol, where ATF4 expression is upregulated and active [43] (Figure 4F). We also found that Fosl2 RNA is up-regulated by cholesterol relative to control in our transcriptomic data (10 to 14 rpkm at 24 h. and 9 to 14 rpkm at 48 h; 2-tailed *t*-test *P* < 0.05), consistent with its role in regulating the VSMC transcriptional response to cholesterol.

### Cholesterol Loading of VSMCs In Vitro Fails to Recapitulate Cell State Transitions Observed in Atherosclerosis whereas oxLDL Macrophages Show Similar Transcriptomes to In Vivo States

**Figure 6.**
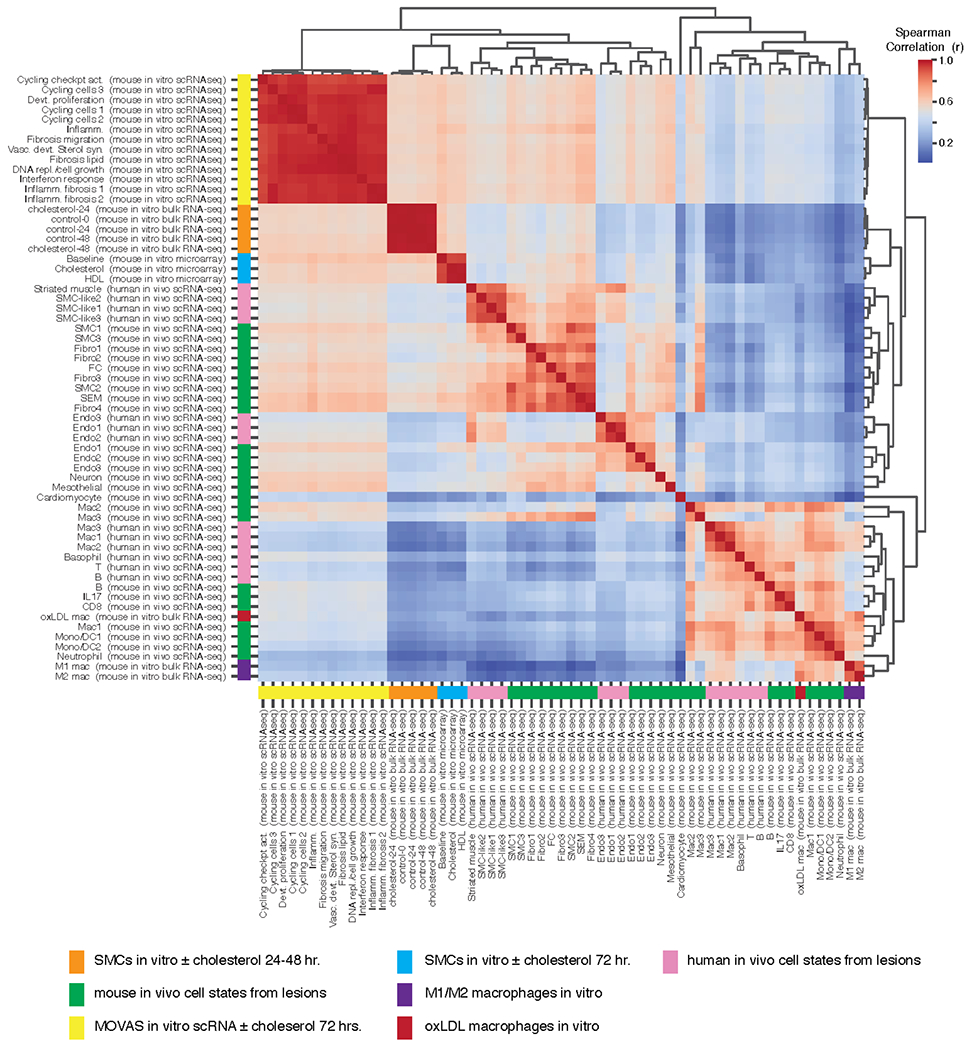
In vitro models of VSMCs and macrophages, with or without pro-atherosclerotic stimuli, fail to segregate with their presumed in vivo counterparts. Pairwise Spearman correlation (given the 2000 most variable genes in the mouse scRNA-seq data) across in vitro and in vivo datasets and/or single cell clusters from mouse and human atherosclerotic lesions are shown.

A major motivation to transcriptionally characterize VSMCs in vitro under untreated and cholesterol-treated conditions was to compare these transcriptomic signatures to VSMC lineage positive cells from atherosclerotic lesions in vivo. In this way, we could determine whether the in vitro system was a useful model with which to study changes that occur in atherosclerosis. Our approach was to compare the average expression profiles of major cell states observed in murine atherosclerotic lesions (Figure 1) to expression profiles measured in vitro VSMCs ± cholesterol using Spearman correlation (using the top 2000 most variably expressed genes from the in vivo scRNA-seq data). Clustering of these pairwise relationships showed that all in vitro VSMC conditions were more similar to one another than to any in vivo mouse cell clusters (Figure 6; in vitro in orange; in vivo in green side bar). These data suggest that up to 48 h cholesterol loading in vitro is not sufficient to reproduce cell state transitions observed in vivo; yet, we questioned whether or not longer cholesterol exposure or differences in platforms measuring expression (i.e., bulk vs scRNA-seq) were responsible for differences to in vivo. We therefore retrieved a public 72 h. cholesterol loaded VSMC dataset [44] as well as collected scRNA-seq data in the mouse VSMC MOVAS cell line ± cholesterol for 48 and 72 h. Interestingly, we observed 13 cell clusters from the scRNA-seq in vitro experiment, suggesting notable heterogeneity exists in vitro, especially in the proliferative and fibrotic and inflammatory states (Supplementary Figure S6). Nonetheless, neither the bulk nor the scRNA in vitro cell profiles clustered with in vivo cell states from lesions (Figure 6; 72 h. bulk in blue and MOVAS scRNA-seq in yellow side bars).

Next, we sought to understand if any in vitro cell model would cluster with in vivo cell profiles, and so we retrieved two datasets of mouse thioglycolate-elicited peritoneal macrophages that were exposed in vitro to oxidized LDL (oxLDL), or to lipopolysaccharide and Interferon gamma (to mimic M1 polarization), or to Interleukin 4 (to mimic M2 polarization) [39]. The macrophages treated with oxLDL are denoted in Figure 6 by red side bar, and M1 and M2 macrophages are denoted by purple. We found that the oxLDL treated macrophage profiles closely clustered with the Mac1 and Mono/DC1/2 in vivo clusters from mouse lesions, suggesting this model closely resembles in vivo macrophage biology in lesions. Lastly, we utilized this comparison approach to test how well human and mouse in vivo cell clusters from scRNA-seq data resembled one another. For this, we retrieved scRNA-seq data from human carotid lesion endarterectomy samples [14], performed clustering, and sample annotations similar to the mouse data (Supplementary Figure S7). Clustering of the resulting cell state profiles showed close relationships between human and mouse in vivo data (Figure 6; mouse in green, human in pink). We found that human VSMCs clustered closest to mouse SMCs, Fibros, and SEMs, human and mouse Endos clustered together, and immune cells from each species clustered together as well. One interesting finding from this analysis is that although they do not cluster together, there is correlation between the in vivo mouse Mac3 cluster and the in vivo mouse Fibro4, SEM, and FC clusters (Figure 6), which supports that these cells might be related by lineage in vivo.

This comparison of average expression profiles among several in vitro and in vivo datasets demonstrates that cholesterol treatment of VSMCs in vitro fails to recapitulate the full extent of cell state transitions observed in murine models of atherosclerosis. We sought to test this conclusion using a second analysis of differentially expressed genes that were defined either in vitro or in vivo and compared profiles across datasets. As shown in Supplementary Figure S8 (left), the genes from the four clusters identified in our analysis of in vitro VSMCs ± cholesterol in Figure 4 were not similarly regulated across in vivo mouse cell clusters nor in the scRNA-seq in vitro data. Similarly, gene sets that are differentially expressed between in vivo mouse clusters are not regulated by cholesterol in vitro by analysis of either bulk or scRNA-seq (Supplementary Figure S8, right). Taken together, results from our analyses demonstrate that in vitro cholesterol loading in VSMCs fails to model the VSMC plasticity that is observed in vivo.

## DISCUSSION

With over 70,000 single cell transcriptomes incorporated, this analysis serves as the largest interrogation of VSMC lineage positive cells in atherosclerosis to date. The relatively large number of cells enabled fine-grained clustering of VSMC lineage positive cells, which as a whole underscore the well-appreciated notion that VSMCs are highly plastic in their vascular phenotypes. VSMC lineage-positive cells in atherosclerotic plaques were observed to comprise significant proportions of contractile VSMCs, de-differentiated VSMCs (termed SEMs), fibroblasts, FC, macrophages, and smaller proportions of endothelial and other cell types. While our meta-analysis supported that VSMCs may take on each of these phenotypic states, notable heterogeneity across studies was observed that obscures precise quantification and raises questions as to the sources of variation. In addition, we found that the expressions of many genes were altered by in vitro cholesterol loading with less profound alterations in the histone acetylation profile. Comparison of these signatures with expression changes across in vivo VSMC lineage populations revealed significant discrepancies between in vivo and in vitro, demonstrating an outstanding need for developing cell culture models of VSMC modulation that better capitulate the phenotypic plasticity of VSMCs in atherosclerotic lesions. These points are further discussed below.

The large number of lineage positive VSMCs in SEM, FC, and fibroblast populations in this meta-analysis provides perhaps the clearest delineation between contractile VSMCs, FCs and fibroblasts to date (Figure 1A; Supplementary Figure S8). Therefore, transcripts that distinguish these cell states may serve as valuable phenotypic markers for future studies (Supplementary Table S2). Data presented here supports the hypothesis that VSMCs can transition to either fibroblast-like or FC-like cells but are more likely to transition to FC-like cells than fibroblast-like cells (Figure 2A). The pseudotime analysis performed here in Figure 2C supports the notion that VSMCs can traverse several different trajectories in atherosclerotic lesions with many of these transitioning through the SEM state, which has been proposed to represent a de-differentiated stem-like cell state [14].

We also found in our meta-analysis of lineage traced VSMCs in murine atherosclerotic plaques that as many as 66% of foamy macrophages across all studies examined were VSMC lineage positive (Figure 3), which is consistent with a recent report [9]. Trajectory analysis also revealed one trajectory that traversed from VSMCs to a fibroblast-like state to the Mac2 population and termination in the macrophage state. This result is provocative insofar as it implies a cell state conversion through a proliferative macrophage-like state (Mac2) and final differentiation into macrophage-like cells. Such a process mirrors experimental observations in Rainbow mice that randomly recombine combinations of florescent markers only in the VSMC lineage [45]. In that study, clones of proliferating and migrating VSMCs become ‘synthetic’ and take on foamy appearances in the sub-endothelial locale. The proliferative and macrophage-like signature in Mac2 could also resemble stem-like monocyte cells reported by Lin et al. in lesions in both atherosclerosis progression and regression, with their ultimate phenotypic state likely determined by the microenvironment [46]. We also found that the Mac3 in vivo population has similarities to SEMs and FCs, suggesting that there might be a developmental similarity between them (Figure 6).

An important consideration surrounding the possibility that VSMCs differentiate into macrophages is the marked difference in VSMC-lineage positive macrophage-like cells across studies (Figure 2A, Supplementary Figures S1 and S4). In particular, Wirka et al. [17] and Kim et al. [35] do not report nearly as many VSMC-lineage positive macrophage-like cells as Pan et al. [14] or Alencar et al. [12]. We speculate that technical differences may be the source of this difference, such as mode and/or dose of tamoxifen administration, leaky Cre expression, cell dissociation protocols (with possible biased recoveries), sorting parameters including gating strategies, and downstream processing of samples through data quality control. Because we observe higher ZsGreen transcript among lineage positive macrophages than among lineage negative macrophages in the Pan et al. data, we suggest that auto-fluorescence is not a sufficient explanation for the high number of apparently SMC-derived macrophages observed. Macrophages and/or lipid-laden cells are most variable across studies, which is consistent with such cells being among the most fragile and thus difficult to isolate intact in order to interrogate them using these methods [18]. Studies that overcome these limitations and sources of variability will be of great value to definitively laying this important question to rest.

One of the motivations for this meta-analysis was to enable a comprehensive glimpse into the expression changes that occur to VSMCs in vivo to enable comparison to models of VSMC modulation in vitro. For VSMCs loaded with cholesterol in vitro, we observed robust alterations in transcript regulation with notable induction of the UPR and repression of cholesterol biosynthetic pathway genes (Figure 4). Each of these pathways have been described to be regulated by cholesterol loading, with most work on the UPR in atherosclerosis occurring in macrophages. There, cholesterol loading was shown to induce the UPR in vitro and in vivo leading to macrophage apoptosis [47]. For VSMCs, a recent report used marker genes as evidence that the UPR is similarly activated in VSMCs in atherosclerotic lesions as occurs upon cholesterol loading in vitro [48]. The authors further conclude that activation of the UPR, as elicited by cholesterol, is the direct mechanism of phenotypic modulation; however, our study indicates that the UPR is induced by cholesterol in vitro, but that this induction is insufficient to reproduce the phenotypic states observed in vivo, as perhaps best illustrated by the heat map in Figure 6.

Because of the convenience of assessing chromatin organization in vitro, we also wished to extend our analysis beyond transcriptome profiling. Based on previous experience studying changes in the histone acetylation changes to pro-atherogenic stimuli in endothelial cells [49] and macrophages [50], we interrogated the cholesterol-loaded and control VSMC epigenomes. Despite the >4000 differentially expressed genes across the dataset, we found that the epigenomes were surprisingly similar. Still, we found evidence that VSMCs utilize AP-1, TEAD, and KLF motifs for regulating gene expression with enrichment of motif frequencies for Fosl2 and Atf4 in cholesterol-induced elements (Figure 5).

Finally, our comparison of expression changes across the in vivo populations and in vitro samples revealed considerable dissimilarity in the VSMC cell states, with each data type clustering in their own branches of the dendrogram (Figure 6). More targeted analysis from both the in vitro-defined differential genes and the in vivo-defined differential genes underscored general discordance between datasets (Supplementary Figure S8), which remained dissimilar for longer (72 h) cholesterol treatments. Because it is the rare VSMC in the arterial media that gives rise by clonal expansion to the cells of VSMC origin in the intima [45], we thought bulk RNA-seq on the cholesterol-loaded VSMC may have obscured changes in a small subpopulation of cells relevant to the in vivo setting. Thus, we collected and analyzed a scRNA-seq dataset from a mouse VSMC cell line loaded with cholesterol or treated with buffer. Interestingly, we did observe multiple cell populations for cholesterol loaded VSMCs in culture (Supplementary Figure S6), and although none were very similar to in vivo transcriptomes, this finding underscores that phenotypic transitions may exist for some cells in culture and that single cell approaches may be necessary to capture the molecular basis for this heterogeneity.

Although we were able to resolve multiple cell populations using scRNA-seq, that we still were not able to qualitatively recapitulate the in vivo findings, particularly the transitional state, may reflect that either the cholesterol-loading methods (cyclodextrin complexes) was unphysiologic or that three-dimensional co-culture systems with other cell types are needed to mimic the complex environment in an atherosclerotic plaque. That macrophages loaded with oxLDL did invade an in vivo macrophage branch of the dendrogram (Figure 6) supports the notion that some in vitro models are more appropriate for modeling in vivo than others. Interestingly, M1 and M2 polarized macrophages in culture were less similar to in vivo gene signatures than oxLDL macrophages, indicating that pure M1/M2 responses are not as important as the effect of oxLDL for modeling the transcriptional state of atherosclerotic macrophages. We also note that oxLDL treatment of VSMCs has not, to our knowledge, been investigated using scRNA-seq; we suggest that such an experiment may shed light on microenvironmental cues for VSMC phenotypic plasticity.

Lastly, we tested and confirmed that signatures of cell populations from human atherosclerotic lesions resembled those discovered in mouse (Figure 6). Taken together, these data suggest that in vitro models of VSMC phenotypic transitions, which are valuable for mechanistic interrogations, need refinement, but also that continued studies of mouse models are likely to yield clinically relevant findings. Greater clarity into the microenvironmental signals and cell-cell interactions that govern phenotypic switching should propel this area of research.

## CONCLUSIONS

Based on the meta-analysis of multiple mouse studies with lineage marked VSMC and comparisons to data from human plaques and VSMC in culture, we offer the following conclusions: (1) There is broad agreement over the plasticity of VSMC in atherosclerosis, with a convergent finding that there is a set of cells called SEM, which has been proposed to represent an intermediate VSMC phenotypic switching state [14] that serves as a platform to lead to a variety of ultimate fates; (2) transcriptome-based cell type characterization is variable across the studies. In particular, while there was clear evidence for macrophage-like cells originating from VSMC in the aggregated data, there was a wide quantitative range between studies. In addition, based on trajectory analysis, there was a path from contractile VSMC to macrophage-like cells by way of a proliferative cell cluster, but it remains incomplete what are the intermediate states a VSMC must progress through; (3) The mouse and human data have many similarities, supporting the continued use of mouse models to draw inferences about VSMC fates in human atherosclerosis; and, (4) though cholesterol-loading of VSMC in vitro resulted in thousands of DEGs, the analyses of either bulk RNA-seq or scRNA-seq data did not reveal striking resemblances to the meta-analysis of the in vivo data. Thus, more progress in model development will be needed before the clinical relevance of results from in vitro systems can be assumed.

## Supplementary Material

Supplementary Figures

Supplementary Tables

## Figures and Tables

**Table 1.** Studies of SMC-lineage tracing and scRNA-seq in murine atherosclerosis analyzed into our meta-analysis.

Publication	Model	Duration of diet (weeks)	Artery section(s)	PMID	GSE
Alencar et al. *Circulation* 2020 [12]	ApoE^−/−^	18	microdissected BCA (lesions); aorta healthy control	32674599	GSE150644
Pan et al. *Circulation* 2020 [14]	ApoE^−/−^	0, 8, 16, 22	ascending aorta, BCA, thoracic aorta	32962412	GSE155513
Pan et al. *Circulation* 2020 [14]	LDLR^−/−^	0, 8, 16, 26	ascending aorta, BCA, thoracic aorta	32962412	GSE155513
Wirka et al. *Nature Medicine* 2019 [17]	ApoE^−/−^	0, 8, 16	aortic root, ascending aorta	31359001	GSE131780
Kim et al. *Circulation* 2020 [35]	ApoE^−/−^	16	aortic root, ascending aorta	32441123	GSE150768

## Data Availability

New data produced in the course of this study are publicly available in the NIH Gene Expression Omnibus (GEO) repository under accession GSE174029 and GSE168520. Previously reported data are all publicly available through GEO in the accessions provided in Supplementary Table S1.

## ABBREVIATIONS

VSMC: vascular smooth muscle cell
scRNA-seq: single cell RNA sequencing
UMAP: uniform manifold approximation and projection
FACS: fluorescence-activated cell sorting
