# Supplementary material for: Meta-Analysis of Smooth Muscle Lineage Transcriptomes in Atherosclerosis and Their Relationships to In Vitro Models
Source: Immunometabolism. Author manuscript; Available in PMC 2021 Jun 25. (PMC8232871; doi:10.20900/immunometab20210022)
Supplement: Supplementary Figures [file NIHMS1707691-supplement-Supplementary_Figures.pdf]

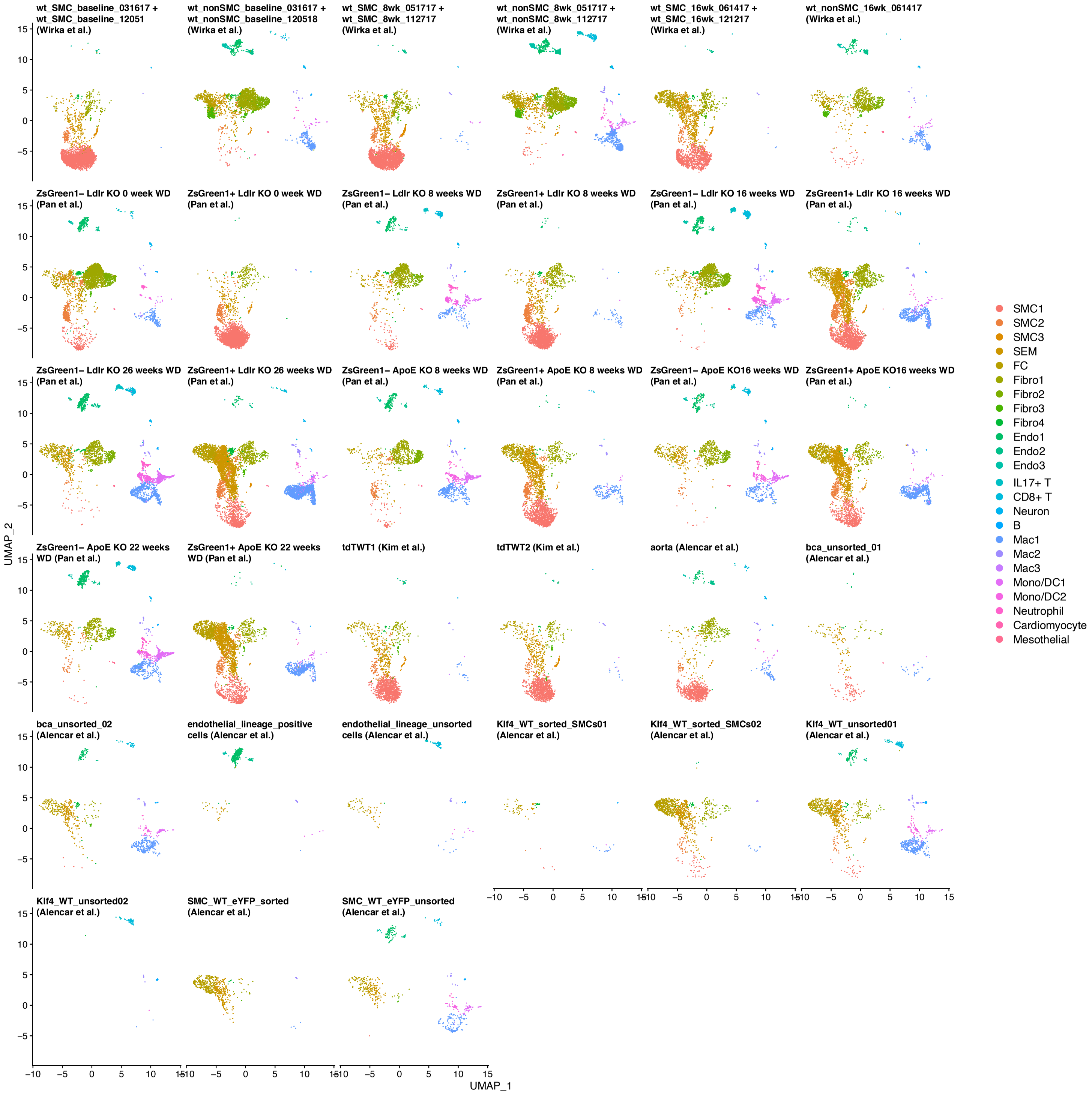

Supplementary Figure S1. UMAP representation of cells across samples and studies. UMAP of single cells from 33 scRNA-seq sample conditions across 4 studies are colored by cluster and split by experimental condition (with study annotated).

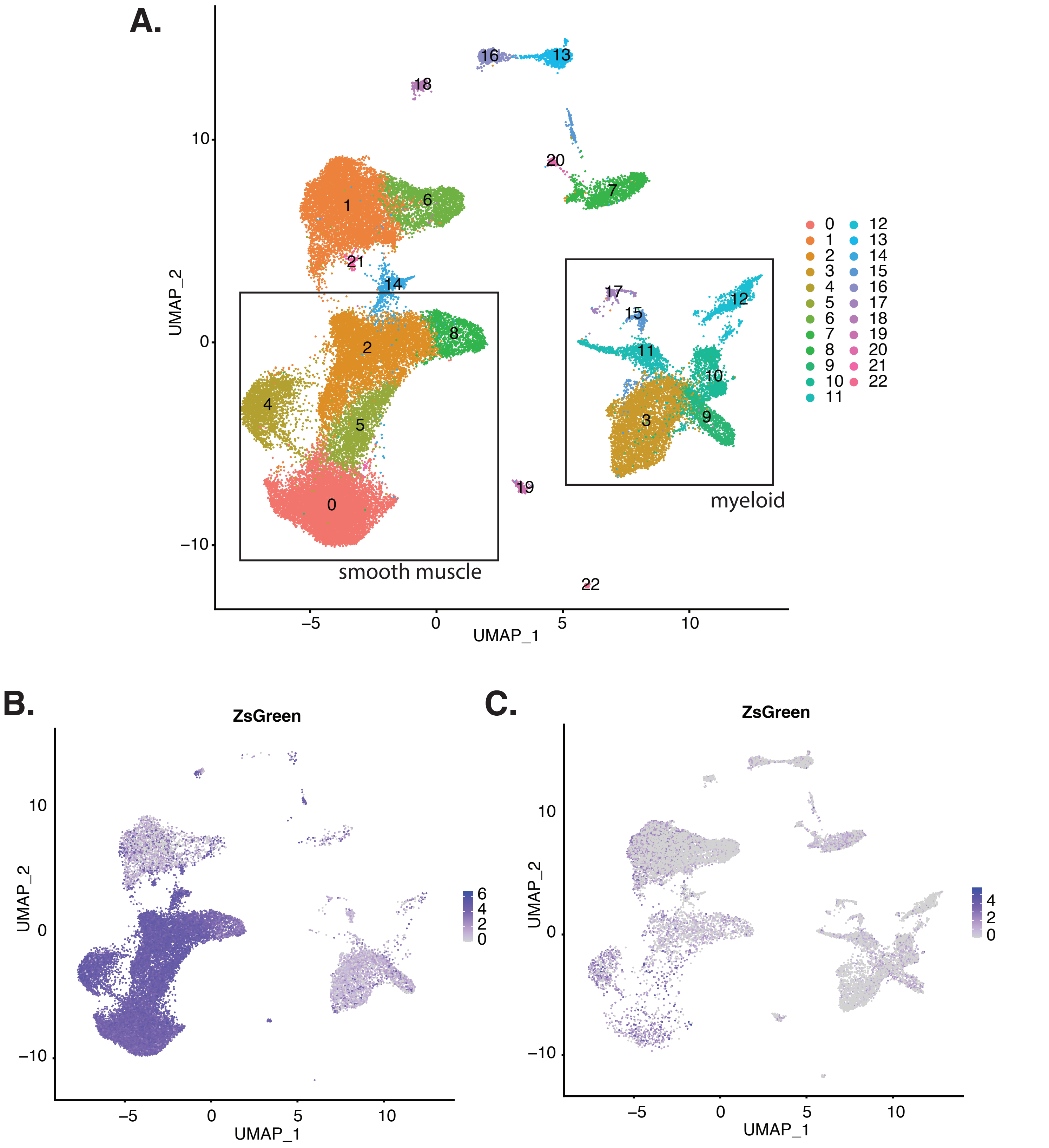

Supplementary Figure S2. ZsGreen transcript expression in Pan et al. lineage positive and lineage negative cells. UMAP of single cells from Pan et al. with SMC-like and myeloid clusters indicated (A). ZsGreen expression is shown in lineage positive sorted (B) and lineage negative sorted (C) cells.

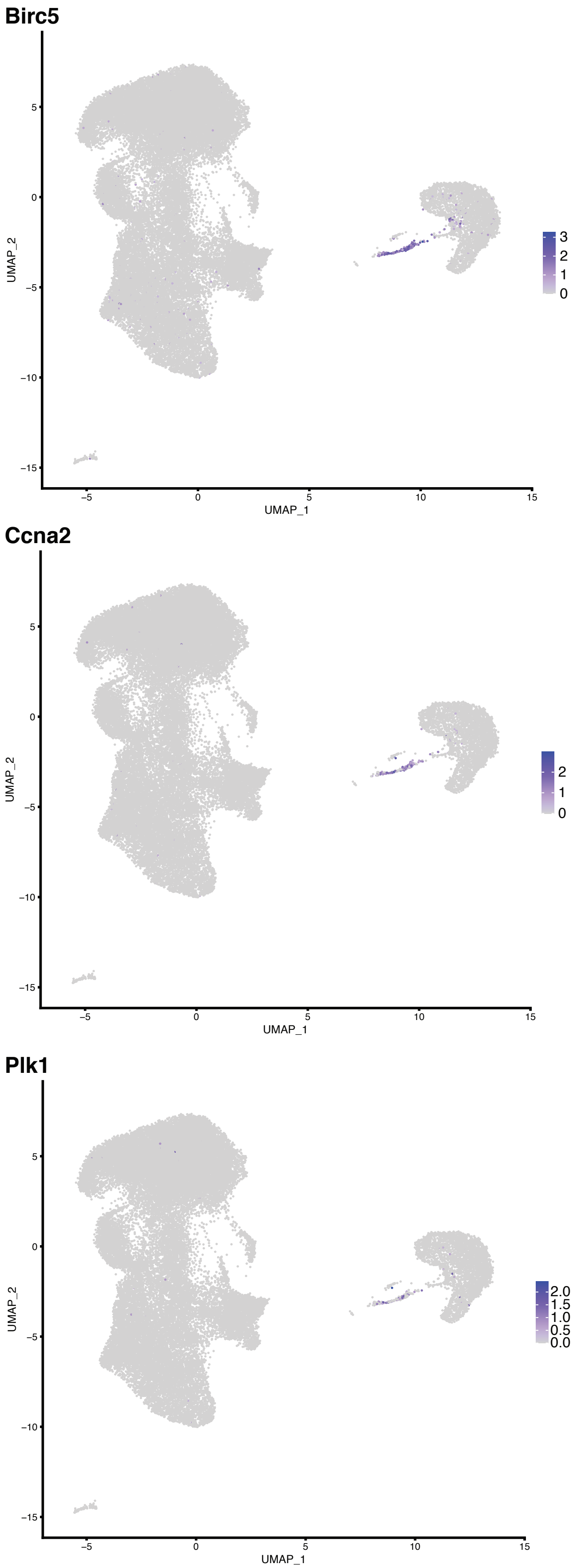

Supplementary Figure S3. Expression of proliferative marker genes in potential SMC-macrophage intermediate cells. Expression of various proliferative marker genes Birc5, Ccna2, and Plk1 are shown in a cluster of cells that may be an intermediate phenotype between SMC and macrophage.

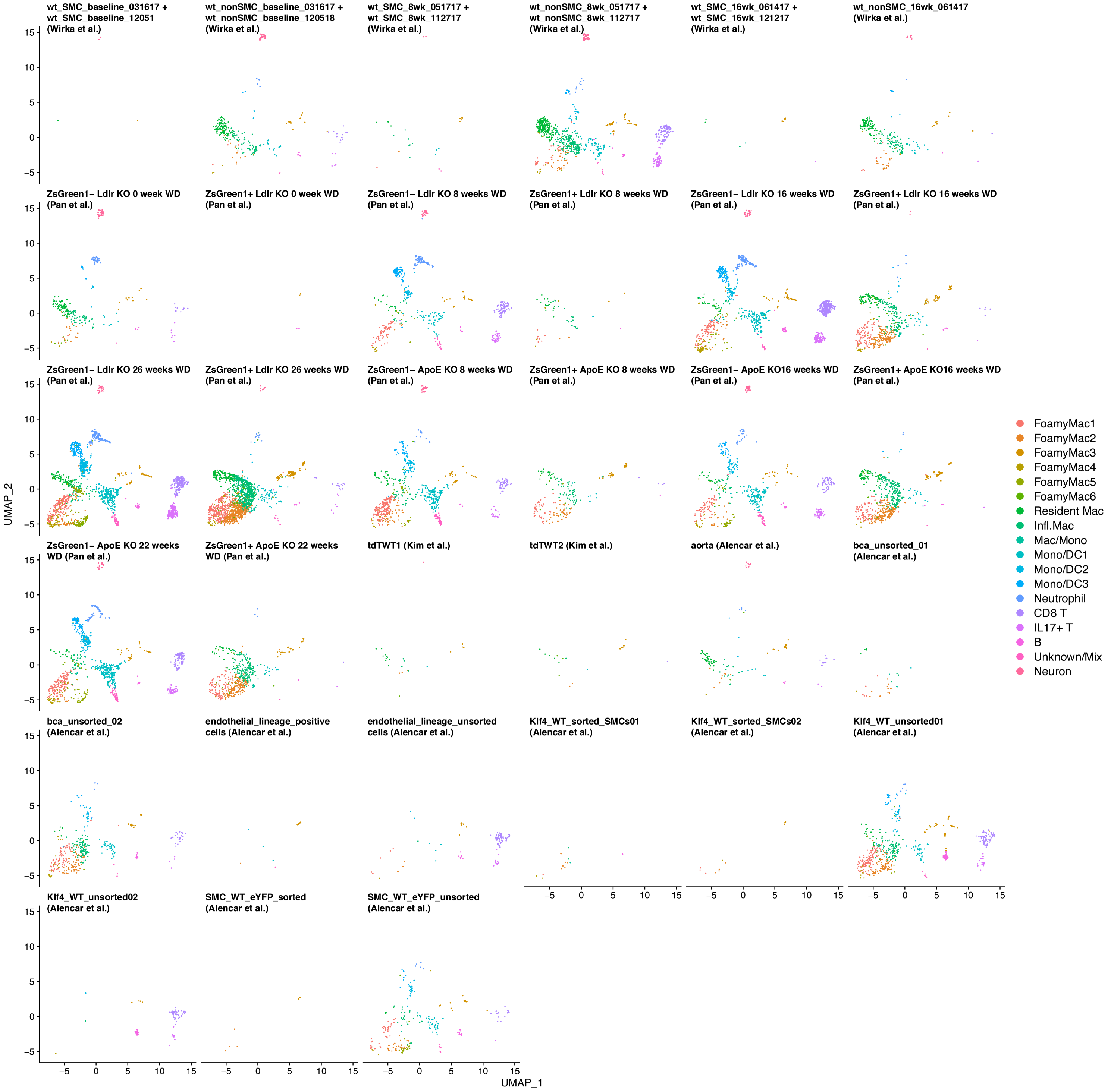

Supplementary Figure S4. UMAP representation of cells across samples and studies for immune cell re-clustering. UMAP of single cells from 33 scRNA-seq sample conditions across 4 studies are colored by cluster and split by experimental condition (with study annotated) after re-clustering of immune cells.

A.

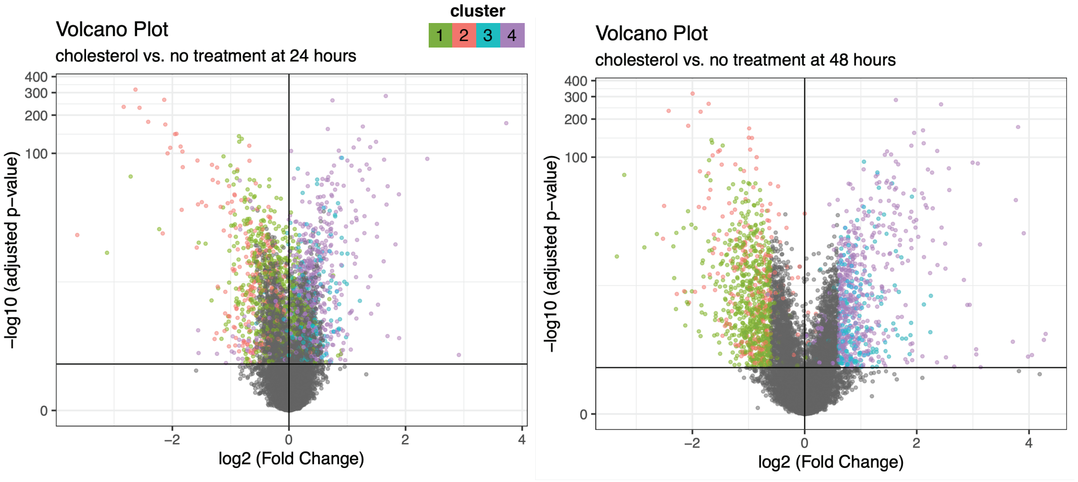

B.

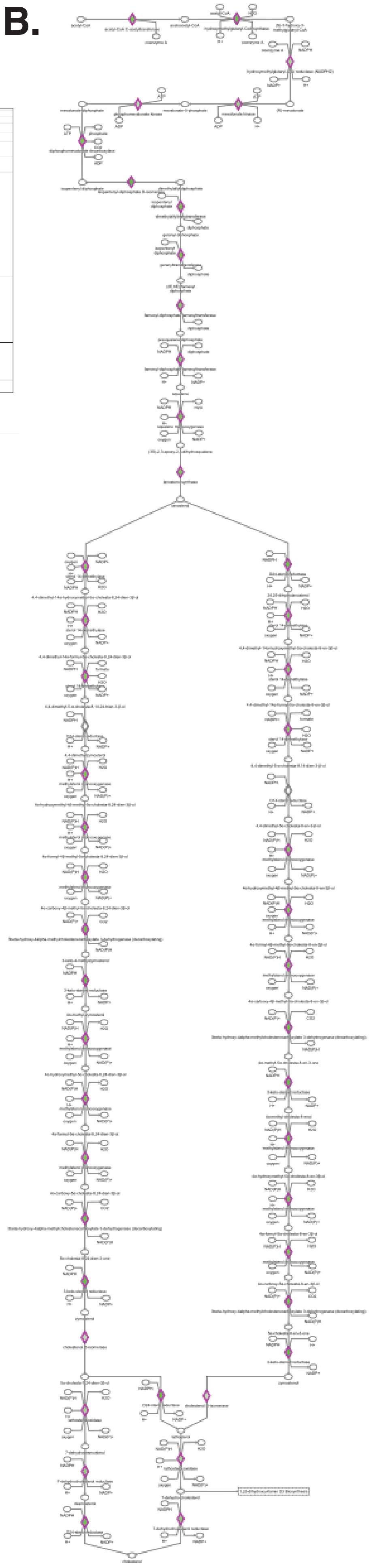

C.

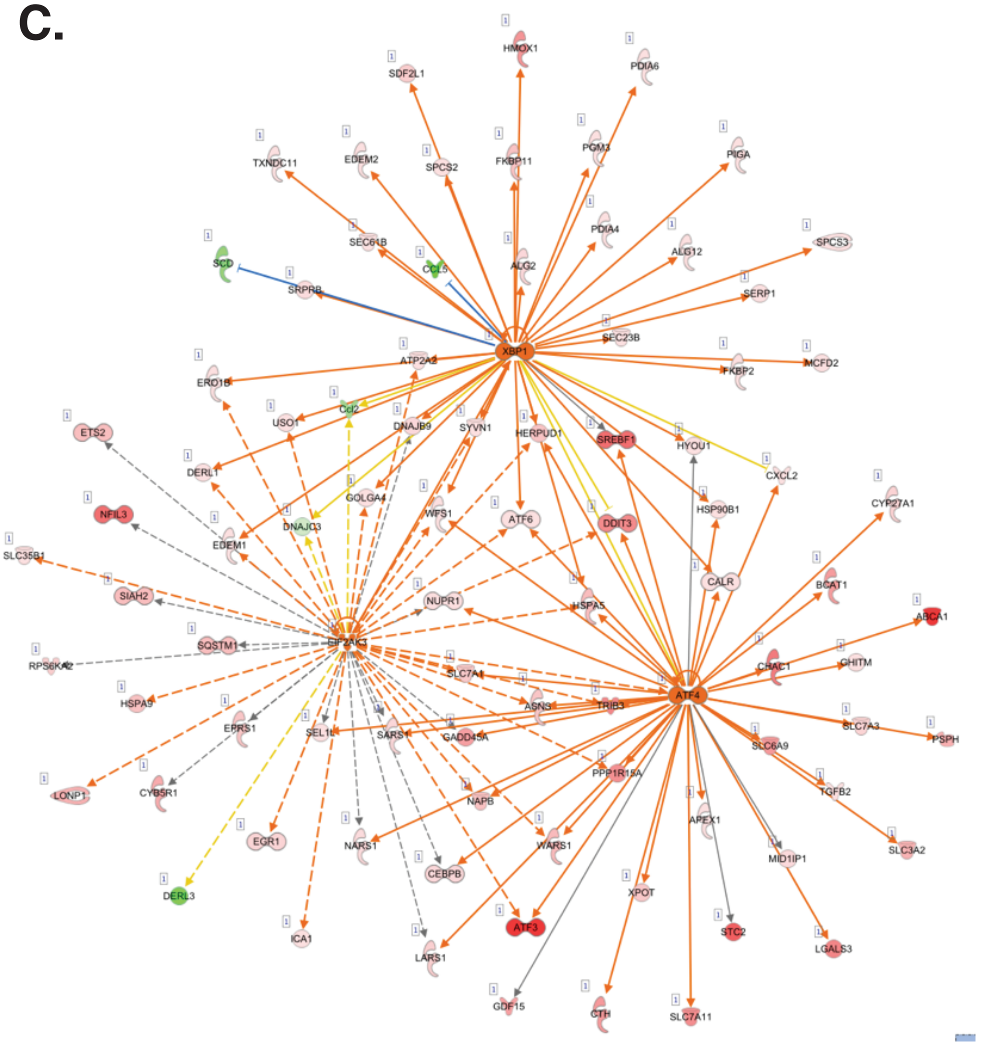

Supplementary Figure S5. Genes and pathways regulated by cholesterol loading in VSMC cultures. (A) Volcano plots showing the statistical significance (y-axes; -log adjusted P-value) versus log2 fold change (x-axes) of transcripts differentially regulated by cholesterol at 24 hours (left) and 48 hours (right). (B) The Superpathway of Cholesterol Biosynthesis is shown as output from Ingenuity with genes regulated by cholesterol highlighted in red. (C) Cholesterol regulated genes in the UPR pathway are shown as output from Ingenuity as a network, with genes upregulated by cholesterol in orange and red or downregulated by cholesterol in green. Lines with arrows indicate curated relationships that have been described in the literature.

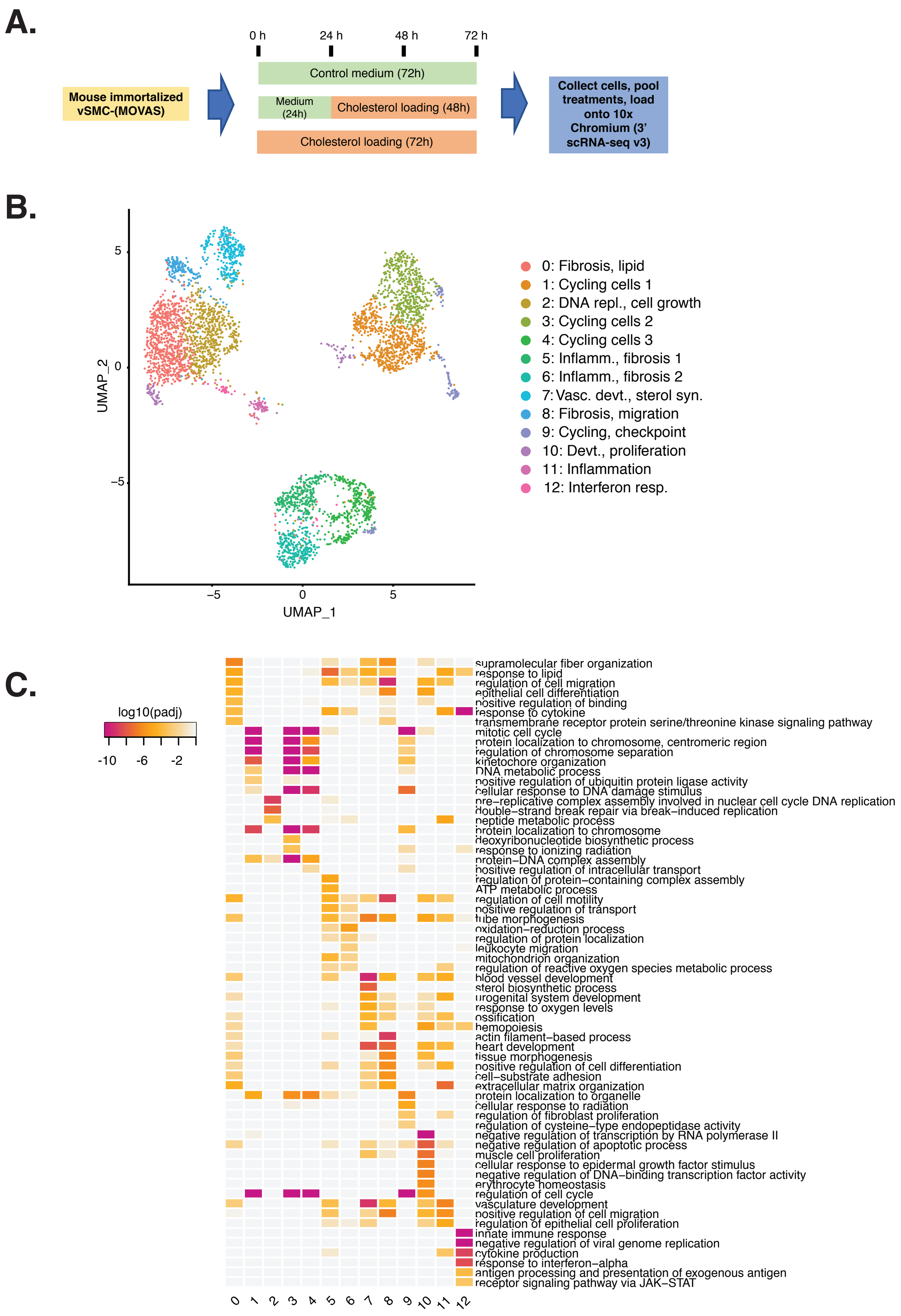

Supplementary Figure S6. Cell culture cholesterol treatment of MOVAS cells with scRNA-seq reveals multiple populations. (A) The experimental design of cholesterol loading and cell collection. (B) UMAP representation of resulting cell clusters. (C) Annotation of clusters using pathway ontologies.

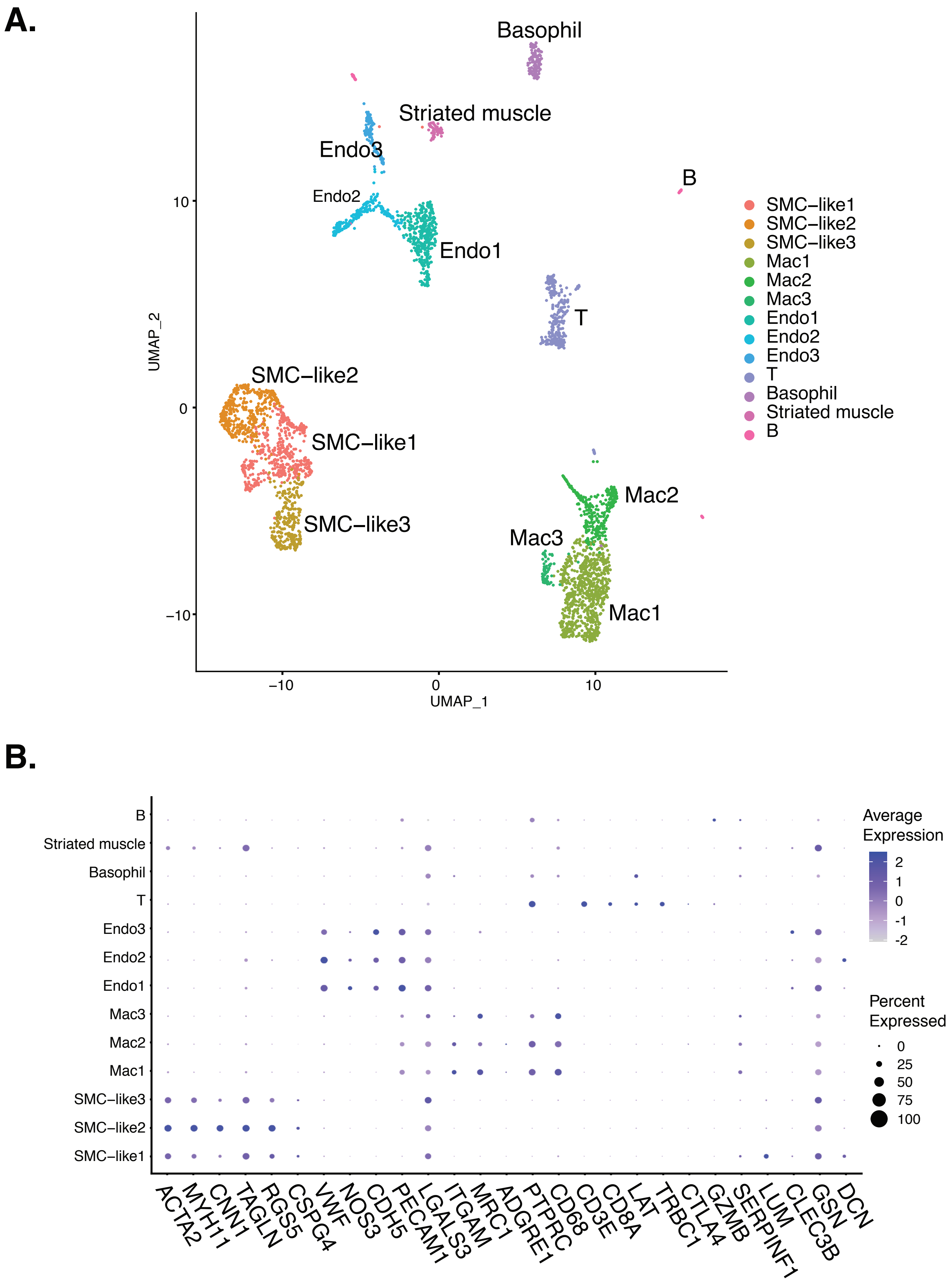

Supplementary Figure S7. Single cell analysis of human carotid lesions reveals multiple cell clusters. UMAP of single cells from scRNA-seq human carotid artery sample (carotid1 from Pan et al.) colored by cluster and annotated.

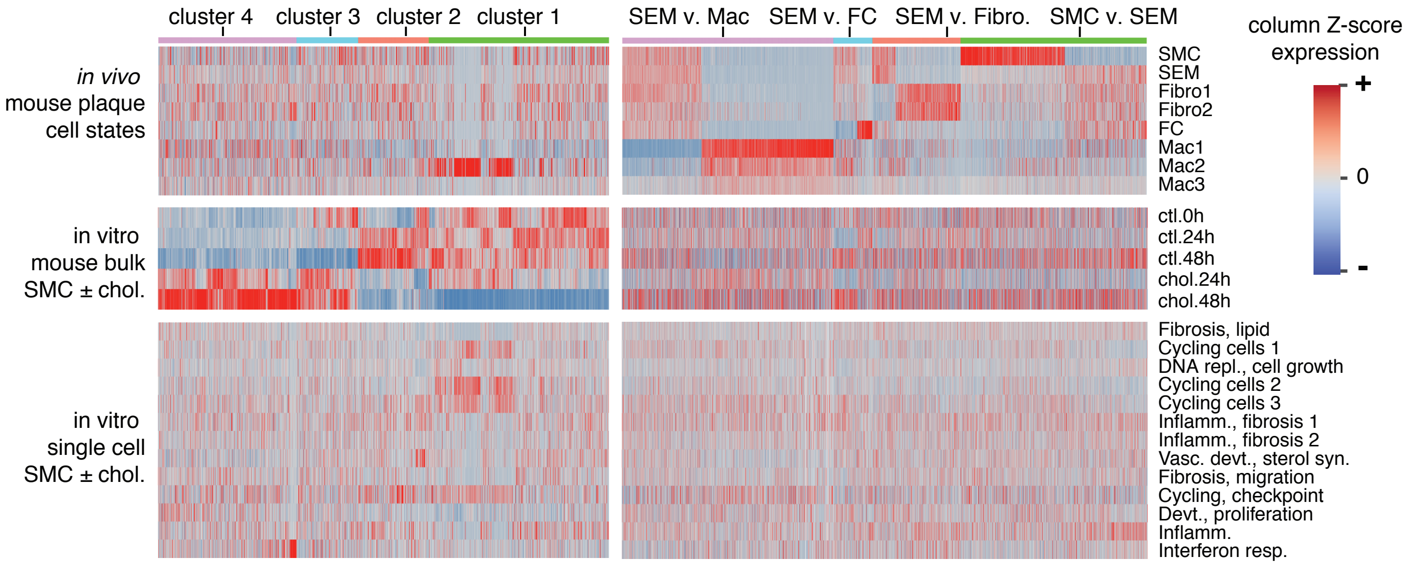

Supplementary Figure S8. Targeted analysis of in vitro and in vivo defined gene sets reveals discordance between transcript profiles. The left plots show expression profiles for the 4 clusters (top colored bars; from Figure 4) of cholesterol regulated genes that were defined in vitro bulk RNA-seq. The right plots show expression profiles for 4 sets of differentially expressed genes between the following in vivo mouse lesion clusters: SMC vs Mac, SEM vs FC, SEM vs Fibro, and SMC vs SEMs. The topmost heatmap shows expression of genes (along x-axis, but too many to label) for mouse in vivo clusters, the middle heatmap shows expression of the bulk in vitro samples, and the bottom heatmap shows expression measured by scRNA-seq in the clusters found in MOVAS cell cultures.
